# Single-cell profiling and genetic regulation of alternative polyadenylation in the human brain

**DOI:** 10.1038/s41588-026-02758-w

**Published:** 2026-09-30

**Authors:** Jiuhong Nan, Carles A. Boix, Shaohui Shi, Xiaoxi Fan, Jiacheng Ni, Kai Wang, Xiaoyu Shuai, Ke Ding, Puqi Wu, Yao An, Na Sun, Lei Hou, Kexuan Chen, Xianpei Huang, Chengyu Li, Leyla Akay, Kate Louderback, Hiba Nawaid, Yongjin P. Park, Xudong Fu, Chong Liu, Xiaoyu Li, Yafei Yin, Wei Mo, Zhanghua Yang, Weirui Ma, Xinyang Hu, David A. Bennett, Manolis Kellis, Li-Huei Tsai, Shamil Sunyaev, Jingyun Li, Xushen Xiong

**Affiliations:** 1 The Second Affiliated Hospital & Liangzhu Laboratory, Zhejiang University School of Medicine, Hangzhou, China; 2 State Key Laboratory of Transvascular Implantation Devices, The Second Affiliated Hospital, Zhejiang University School of Medicine, Hangzhou, China; 3 Department of Biomedical Informatics, Harvard Medical School, Boston, MA USA; 4 The Broad Institute of MIT and Harvard, Cambridge, MA USA; 5 Zhejiang Key Laboratory of Molecular Cancer Biology, Life Sciences Institute, Zhejiang University, Hangzhou, China; 6 Department of Neurosurgery of Second Affiliated Hospital and School of Brain Science and Brain Medicine, Zhejiang University School of Medicine, Hangzhou, China; 7 Liangzhu Laboratory, MOE Frontier Science Center for Brain Science and Brain-machine Integration, State Key Laboratory of Brain-machine Intelligence, Zhejiang University, Hangzhou, China; 8 NHC and CAMS Key Laboratory of Medical Neurobiology, Zhejiang University, Hangzhou, China; 9 Sir Run Run Shaw Hospital, Zhejiang University School of Medicine, Hangzhou, China; 10 Department of Psychiatry and Department of Immunology of Sir Run Run Shaw Hospital, Zhejiang University School of Medicine, Liangzhu Laboratory, Zhejiang University, Hangzhou, China; 11 Department of Biochemistry and Department of Gastroenterology of the Second Affiliated Hospital, Zhejiang University School of Medicine, Hangzhou, China; 12 Computer Science and Artificial Intelligence Lab, Massachusetts Institute of Technology, Cambridge, MA USA; 13 Department of Medicine, Biomedical Genetics Section, Boston University, Boston, MA USA; 14 Picower Institute for Learning and Memory, Department of Brain and Cognitive Sciences, Massachusetts Institute of Technology, Cambridge, MA USA; 15 Department of Pathology and Laboratory Medicine and Department of Statistics, University of British Columbia, Vancouver, British Columbia Canada; 16 Rush Alzheimer’s Disease Center, Rush University Medical Center, Chicago, IL USA

**Keywords:** Gene regulation, Data mining

## Abstract

Alternative polyadenylation (APA) has a central role in regulation of the fate of mRNA and exhibits high variability in the brain and in neurons. However, brain APA regulation has not been mapped at cell type resolution, and the genetic control of APA in brain cell types and its contribution to genetic disease have remained unclear. Here we report a single-cell atlas of APA of the aged human brain across 2 million cells from 379 human postmortem brains across individuals with and without Alzheimer’s disease (AD). We show that APA provides an independent mechanistic layer for understanding gene regulatory changes in AD, as genes with APA alterations are distinct from those with expression changes but often converge to similar pathways, including microglial activation and microtubule transport in neurons. We integrate APA variation with whole-genome sequencing to identify cell-type-resolved 3′ untranslated region quantitative trait loci (3′aQTLs) for 4,288 genes. We find that 3′aQTLs preferentially colocalize with pQTLs over eQTLs. Across 17 brain traits and diseases, we identify 168 GWAS loci dependent on 3′aQTLs, of which only 17.5% are shared with eQTLs. These include *PLEKHA1* and *APOC2* for AD, *PAK6* and *AP3B2* for schizophrenia, *NDUFA13* for bipolar disorder, *MTCH2* for multiple brain traits, and *SNCA*, the top locus in Parkinson’s disease. We show that 3′ untranslated region choice alters *SNCA* mRNA localization within the cell, consistent with the observation that the *SNCA* risk locus acts in an eQTL-independent manner in neurons and oligodendrocytes. Our results provide a cell-type-specific foundation to interrogate the *cis* and *trans* regulation of APA and to understand the contribution to disease heritability of this key post-transcriptional layer.

## Abstract (web summary)

This study provides a single-cell atlas of 3′ untranslated region regulation in the human brain, pointing to molecular mechanisms for genetic variants contributing to Alzheimer’s disease, Parkinson’s disease and other brain disorders beyond gene expression.

## Main

The choice of 3′ untranslated region (UTR) through alternative polyadenylation (APA) is an essential molecular step in mRNA maturation^1–4^. It plays a central part in regulation of the fate of RNA molecules, as sequence, length, structure and binding sites in alternative 3′ UTRs directly affect translation, transcript localization and RNA stability^5–11^. Known APA regulators include proteins of the cleavage and polyadenylation specificity factor and cleavage stimulation factor families, but no full compendium is available^1,5,6,12,13^. Recent and widespread use of 3′-end-biased single-cell RNA sequencing (RNA-seq), which directly captures the polyadenylation sites (pA sites) of detected transcripts, represents a unique opportunity to interrogate the cell-type-resolved APA landscape in health and disease^1,14–18^. In particular, cell-type-specific APA is crucial in the brain, as highly structured neurons rely on precise localization of mRNAs to distal dendrites and synapses^1,2,19,20^. Recent work has implicated altered and cryptic 3′ UTR usage in neurodegeneration^21–24^, yet there is a lack of a comprehensive cell-type-resolved landscape of APA in the human brain and its alteration in disease.

We also have an incomplete understanding of the genetics underlying 3′ UTR choice at the cell type level. Although hundreds of 3′ UTR quantitative trait loci (3′aQTLs) have been identified in bulk tissues^25–28^, single-cell mapping could help us to understand cell-type-specific 3′ UTR regulatory mechanisms. In addition, 3′aQTLs could help us to interpret unexplained disease loci from genome-wide association studies (GWAS), especially given the dearth of colocalization between expression QTLs (eQTLs) and GWAS variants^29–31^. In particular, 3′aQTLs may capture both eQTL-dependent loci affecting mRNA stability and eQTL-independent loci with effects on translation and mRNA localization^30,31^.

Here we report a single-cell APA atlas covering 30,000 pA sites across 2 million nuclei from 379 human postmortem brains from individuals with and without Alzheimer’s disease (AD). We report specific and strong APA differences in late AD, underlying key AD pathways including microglial activation and microtubule-related transport. APA and expression changes in AD were largely orthogonal but often converged on the same pathways. We identified 4,288 genes with genetically regulated APAs (g-APAs), whose 3′aQTLs colocalized more frequently with protein QTLs (pQTLs) than eQTLs. Our single-cell 3′aQTLs explained 168 GWAS loci in 17 brain traits, linking noncoding risk variants to 3′ UTR choice. Notably, we show how 3′aQTLs can drive disease risk both through protein abundance and other nonexpression mechanisms such as mRNA localization. Together, our results provide a cell-type-resolved regulatory map of 3′ UTR choice, paving the way for study of development and disease mechanisms and 3′ UTR-targeted therapeutics.

## Results

### A single-cell mRNA polyadenylation atlas of human brain

**Fig. 1:**
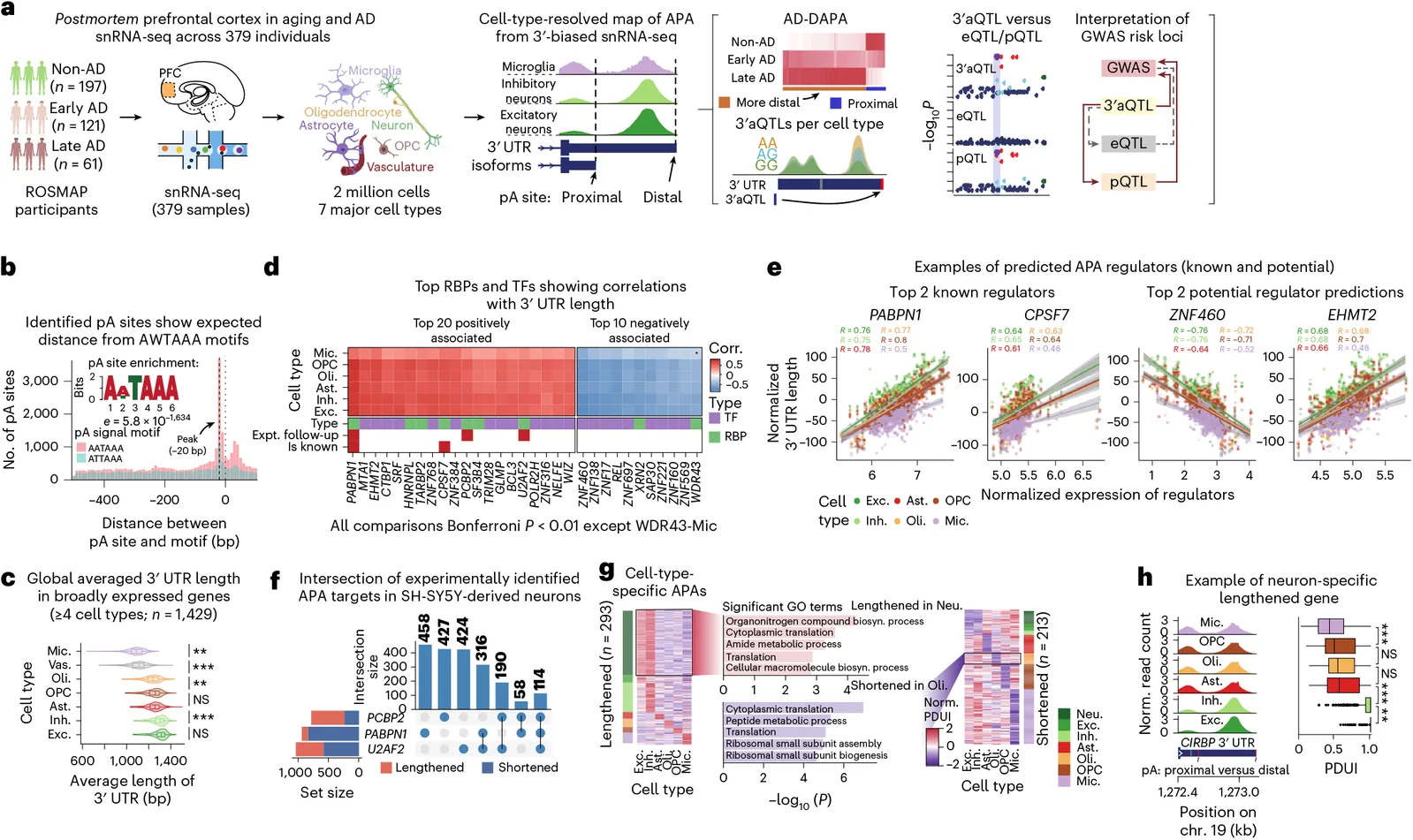
A cell-type-resolved APA landscape of the human brain. **a**, Overview of the study, including data collection, APA quantification and analyses, 3′aQTL identification and GWAS disease interpretation. **b**, Distribution of distances between the known polyadenylation signal (AWTAAA motif) and the identified pA sites. **c**, Average 3′ UTR length of broadly expressed genes (genes expressed in no fewer than four of the seven major cell types; *N* denotes the number of genes used for this analysis) in each major cell type (*P* values were calculated by two-sided Wilcoxon tests, multiple-testing correction was performed using the Holm–Bonferroni method; NS, not significant (*P* ≥ 0.05); **P < 0.01, ***P < 0.001. Exact *P* values: excitatory neurons (Exc.) versus inhibitory neurons (Inh.), *P* = 0.053; Inh. versus astrocytes (Ast.) *P* < 2 × 10^−16^; Ast. versus OPC, *P* = 0.905; OPC versus oligodendrocytes (Oli.), *P* = 0.002; Oli. versus vascular (Vas.) cells, *P* < 2 × 10^−16^; Vas. versus microglia (Mic.), *P* = 0.002. Sample sizes: Exc., *n* = 378; Inh., *n* = 374; Ast., *n* = 378; OPC, *n* = 378; Oli., *n* = 378; Mic., *n* = 371; Vas., *n* = 370). The box shows the 25th–75th percentile, the line shows the median and the whiskers show 1.5× interquartile range (IQR). **d**, Top 30 predicted APA regulators, based on regression between expression of the regulator and the average 3′ UTR length across individuals in each cell type. Regulators are ranked by the magnitude of their average Spearman correlation across cell types. Genes are annotated by whether they are known APA regulators and whether we performed experimental follow-up. **e**, Scatter plots showing the correlation between the expression of top predicted APA regulators and the scaled average 3′ UTR lengths per individual in the major cell types. Left: top two known regulators (*PABPN1* and *CPSF7*); right: top two potential new regulators (*ZNF460* and *EHMT2*), ranked by the correlation between regulator expression and 3′ UTR length. Solid lines represent the predicted mean from the linear regression model, and gray shadows denote the 95% confidence intervals of the regression fit. **f**, UpSet plot showing numbers of shared APA targets across genes based on knockdown experiments in SH-SY5Y-derived neurons. The total number of APA targets is shown on the left, with lengthened and shortened targets separated by color. **g**, Cell-type-specific lengthened (left) and shortened (right) APAs and their functional enrichments (middle) across cell types. Heatmaps show the normalized PDUI across cell types. GO terms enriched for the neuron-lengthened and oligodendrocyte-shortened APA genes are shown (GO enrichment results for lengthened and shortened genes across all cell types are shown in Extended Data Fig. 2d,e). *P* values were calculated using a one-sided hypergeometric test. Terms with raw *P* < 0.01 were considered to be significant. **h**, *CIRBP* as an example of a neuron-specific lengthened APA. Left: raw sequencing coverage tracks in the *CIRBP* 3′ UTR across cell types. Right: boxplot showing the raw PDUI across individuals in each cell type (*P* values were calculated by two-sided Wilcoxon tests; NS, not significant (*P* ≥ 0.05); **P < 0.01, ***P < 0.001. Exact *P* values: Exc. versus Inh., *P* = 7.8 × 10^−3^; Inh. versus Ast., *P* = 7.8 × 10^−72^; Ast. versus Oli., *P* = 1; Oli. versus OPC, *P* = 0.39; OPC versus Mic., *P* = 5.4 × 10^−8^. Sample sizes: Exc., *n* = 371; Inh, *n* = 345; Ast., *n* = 315; OPC, *n* = 324; Oli., *n* = 326; Mic., *n* = 287). The box shows the 25th–75th percentile, the line shows the median and the whiskers show 1.5× IQR. biosyn., biosynthetic; corr., correlation; expt., experimental; Neu., neurons; norm., normalized; PFC, prefrontal cortex. Panel **a** created in BioRender; Xiong, X. https://biorender.com/ijhv96i (2026) and adapted from ref. ^55^, Springer Nature Limited. Source data

**Extended Data Fig. 1:**
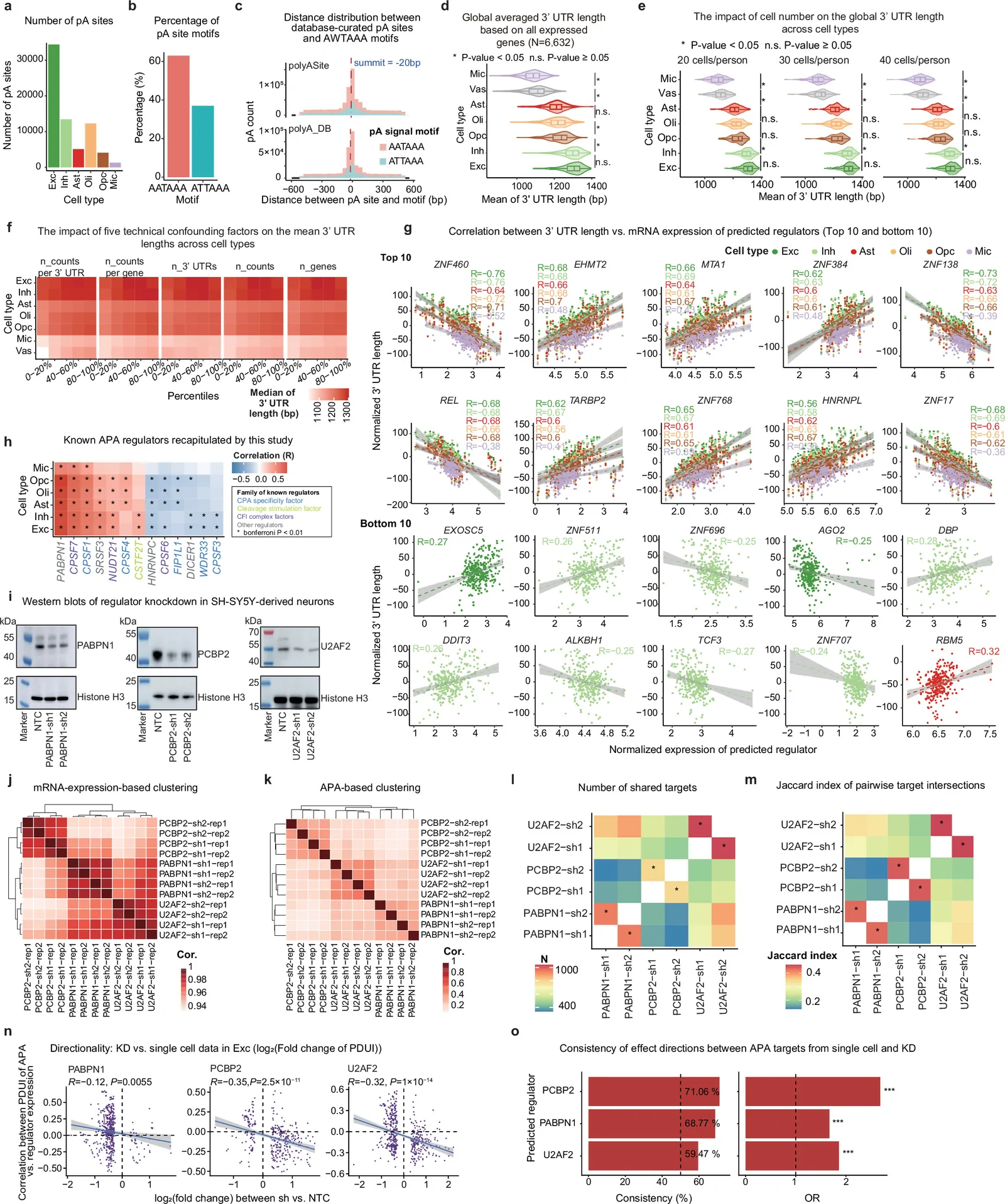
APA landscape across cell types and APA regulator identification, related to Fig. 1. **a**, Number of pA sites identified in each cell type. **b**, Proportion of AWTAAA motifs near identified pA sites. **c**, Relative distance between pA site and nearby AWTAAA motifs in two external databases. **d**, Average 3’UTR length of all expressed genes across cell types. Exact P-values: Exc–Inh=0.14, Inh–Opc<2×10^−16^, Opc–Oli=6.9×10^−4^, Oli–Ast=0.067, Ast–Vas<2×10^−16^, Vas–Mic=1.3×10^−6^; N_Exc_ = 378, N_Inh_ = 374, N_Ast_ = 378, N_Opc_ = 378, N_Oli_ = 378, N_Mic_ = 371, N_Vas_ = 370. **e**, Mean 3’UTR lengths after downsampling to 20, 30 or 40 cells per individual (N = 136, 161 and 184, respectively). Exact P-values of 20-cell: Exc–Inh=0.19, Inh–Opc<2×10^−16^, Opc–Oli=0.19, Oli–Ast=0.19, Ast–Vas<2×10^−16^, Vas–Mic=0.02; 30-cell: Exc–Inh=0.27, Inh–Opc<2×10^−16^, Opc–Oli=0.19, Oli–Ast=0.27, Ast–Vas<2×10^−16^, Vas–Mic=0.01; 40-cell: Exc–Inh=0.2, Inh–Opc<2×10^−16^, Opc–Oli=0.14, Oli–Ast=0.2, Ast–Vas<2×10^−16^, Vas–Mic=0.002. In **d,e**, P values were calculated by two-sided Wilcoxon tests with Holm–Bonferroni correction. Boxes indicate 25th–75th percentiles, lines show the median and whiskers show 1.5×IQR. **f**, Impact of technical confounding factors on mean 3’UTR lengths. **g**, Top and bottom ten predicted APA regulators ranked by regression coefficients with global 3’UTR length. Lines and shading indicate regression fits and 95% confidence intervals. **h**, Known APA regulators among predicted regulators ranked by average Spearman correlation. Spearman correlation coefficients and corresponding two-sided P values were calculated. **i**, Western blots validating regulator knockdown in SH-SY5Y-derived neurons using two independent shRNAs. **j,k**, Pairwise correlations between replicates based on gene expression (**j**) or PDUI (**k**). **l,m**, Shared APA targets (**l**) and Jaccard indices (**m**) between experiments, with two technical replicates per shRNA; asterisks indicate intersections between biological replicates. **n**, Correlation between knockdown-induced fold changes and effect sizes estimated from regulator-PDUI correlations in excitatory neurons. Lines and shading indicate regression fits and 95% confidence intervals. Spearman correlation coefficients and corresponding two-sided P values were calculated. **o**, Consistency of effect directionalities between APA targets identified from regulator knockdown versus from single-cell brain data (left). Enrichment tests for sign consistency in targets compared to non-targets, using two-sided Fisher’s tests (right) (Exact P-values: PABPN1 = 1.61×10^−8^, PCBP2 = 5.66×10^−18^, U2AF2 = 1.27×10^−12^). Source data

To interrogate mRNA polyadenylation in the human brain at cell type resolution, we analyzed our recent single-nucleus transcriptomics (snRNA-seq) atlas of the ageing brain, which profiled 427 postmortem prefrontal cortex samples from the Religious Orders Study and Rush Memory and Aging Project (ROSMAP)^18^. Of these, we selected only data from the 379 individuals profiled using 10x v.3 chemistry (Supplementary Table 1). We used 3′-biased snRNA-seq to identify more than 30,000 unique pA sites from 2 million cells across 7 major cell types, comprising excitatory neurons, inhibitory neurons, astrocytes, oligodendrocytes, oligodendrocyte progenitor cells (OPCs), microglia and vascular cells (Fig. 1a, Extended Data Fig. 1a, Supplementary Table 2 and Methods). The pA sites showed the expected enrichment of canonical polyadenylation motifs upstream of cleavage sites^16,32–34^ (Fig. 1b and Extended Data Fig. 1b,c). On average, across cell types, we observed significantly longer 3′ UTRs in neurons and shorter 3′ UTRs in microglia, consistent with previous reports^10,35,36^ (Extended Data Fig. 1d–f, Supplementary Note and Methods). These differences were preserved among broadly expressed genes and were robust to cell number and complexity, suggesting that they are primarily driven by the global *trans* context of each cell type (Fig. 1c and Extended Data Fig. 1d–f).

To predict *trans* APA regulators, we identified 374 RNA-binding proteins (RBPs) and transcription factors (TFs) whose expression was correlated with 3′ UTR length across individuals; among these were 13 known APA regulators, including *PABPN1*^1,37^, *CPSF1/4*, *CPSF6/7* and *SRSF3*^38,39^ (Extended Data Fig. 1g,h, Supplementary Table 3 and Methods). The top predicted regulators were both negatively and positively associated with 3′ UTR length and included genes that have been linked to brain diseases, such as *SRF* for neurodegeneration and *HNRNPL* for AD and amyotrophic lateral sclerosis^40–42^ (Fig. 1d,e).

To validate predicted APA regulators, we performed gene knockdown in SH-SY5Y-derived neurons and measured the resulting transcriptomes using RNA-seq (Supplementary Note and Methods). We selected known APA regulator *PABPN1* and two predicted regulators, *PCBP2* and *U2AF2* (Extended Data Fig. 1i). Biological replicates showed consistent mRNA and APA landscapes and highly reproducible APA targets (Extended Data Fig. 1j–m and Supplementary Table 4). We identified 1,044 high-confidence targets for *U2AF2*, 946 for *PABPN1* and 789 for *PCBP2*, with *PABPN1* and *U2AF2* sharing more than 40% of their targets (Fig. 1f). Although *U2AF2* has been implicated in mRNA 3′-end processing^43^, our results further support its role in regulation of APA. Notably, knockdown-induced APA changes showed global consistency with the single-cell data despite the different cellular contexts (Extended Data Fig. 1n–o).

**Extended Data Fig. 2:**
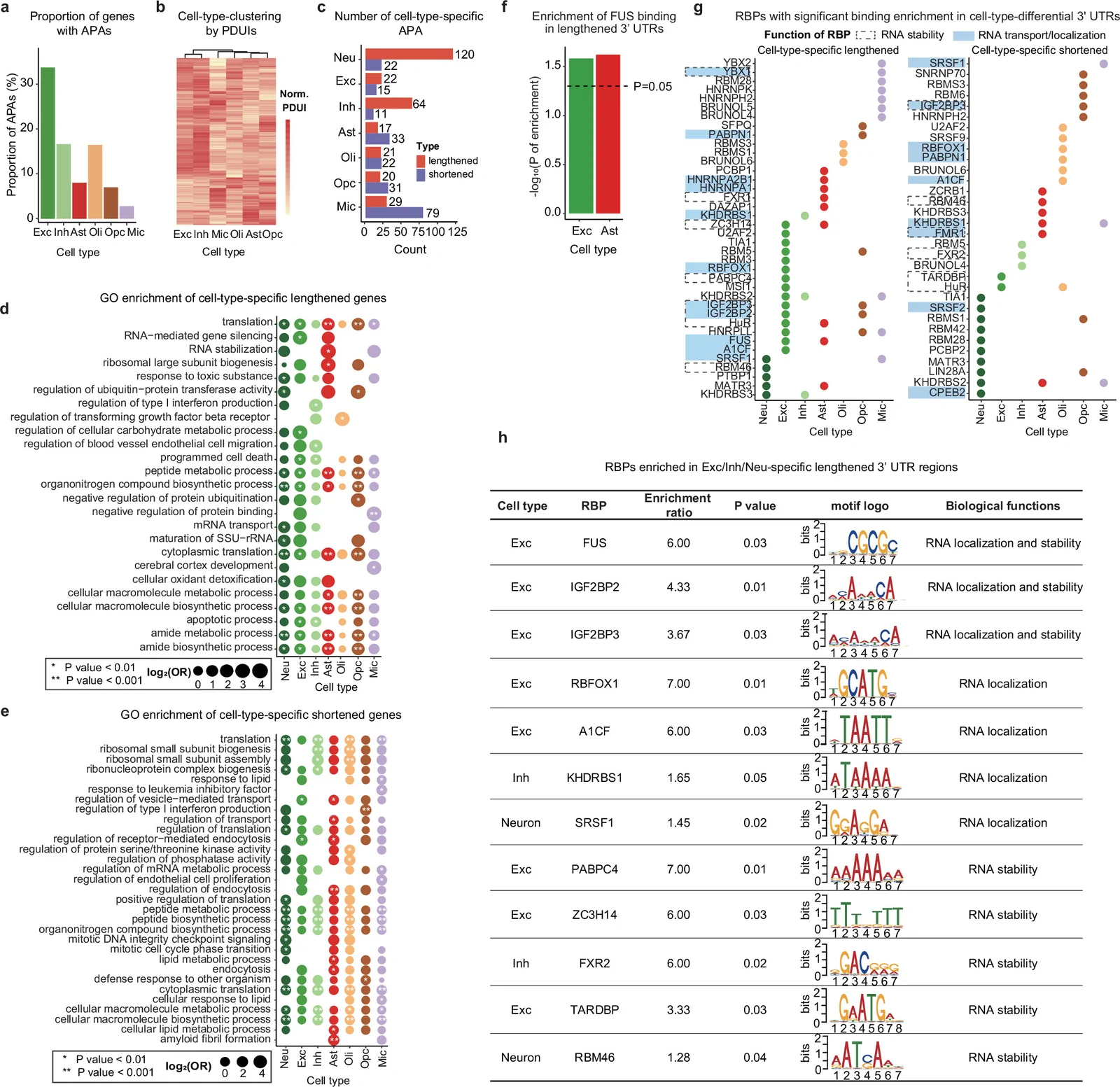
Cell-type-differential APAs and their downstream molecular effects, related to Fig. 1. **a**, Proportion of genes with APAs across the expressed genes in each cell type. **b**, Clustering of samples by cell types using PDUI signals. **c**, Number of cell-type-specific APAs separated by lengthened and shortened 3’UTRs in each cell type. **d**, Functional enrichments of cell-type-lengthened genes across cell types. P value was calculated using a one-sided hypergeometric test. **e**, Functional enrichments of cell-type-shortened genes across cell types. P value was calculated using a one-sided hypergeometric test. **f**, Enrichment of FUS (a known mRNA localization regulator) binding in excitatory- and astrocyte-lengthened 3’UTRs. P value was calculated by SEA using Fisher’s exact test. **g**, RBPs with significantly enriched binding in cell-type-differential 3’UTRs. Known functions of RBPs are indicated by dotted rectangles (involved in RNA stability) and blue shadow (involved in RNA localization). **h**, RBPs whose binding sites were enriched for neuron-specific lengthened 3’UTR regions. The motif and biological function are shown for each RBP. P values were calculated by SEA using Fisher’s exact tests when the primary and control sequences had similar average lengths (differing by<0.01%); otherwise, a Binomial test was applied.

We identified APAs for 2.78–33.76% of expressed genes across cell types (Extended Data Fig. 2a). To quantify APA usage, we aggregated the snRNA-seq data at cell type level for each individual and then calculated percentage of distal pA site usage index (PDUI) values for each gene^25^ (Extended Data Fig. 2b and Methods). We identified 506 genes with differential APA (DAPA) usage between cell types (Fig. 1g, Extended Data Fig. 2c and Supplementary Table 5); these were enriched for various core processes, including translation, mRNA transport and various biosynthetic processes (Fig. 1g and Extended Data Fig. 2d,e). For example, the 3′ UTR of *CIRBP*, whose protein product can bind to 3′ UTRs and regulates APA selection, alternated between almost 100% distal pA site usage in neurons and more than 50% proximal pA site usage in microglia^44,45^ (Fig. 1h).

To explore potential downstream effects, we identified 50 RBPs with binding sites enriched in cell-type-differential 3′ UTRs, including regulators of RNA stability and localization (Extended Data Fig. 2f–h, Supplementary Table 6 and Methods). These included FUS, the binding sites of which were enriched in the lengthened 3′ UTRs of excitatory neurons and astrocytes, consistent with its role in axonal mRNA localization^46,47^ (Extended Data Fig. 2f). Collectively, the regulators and enriched sequence motifs identified here shed light on the *trans* and *cis* regulation of cell-type-differential 3′ UTR usage.

### Differential 3′ UTR choice in AD

**Fig. 2:**
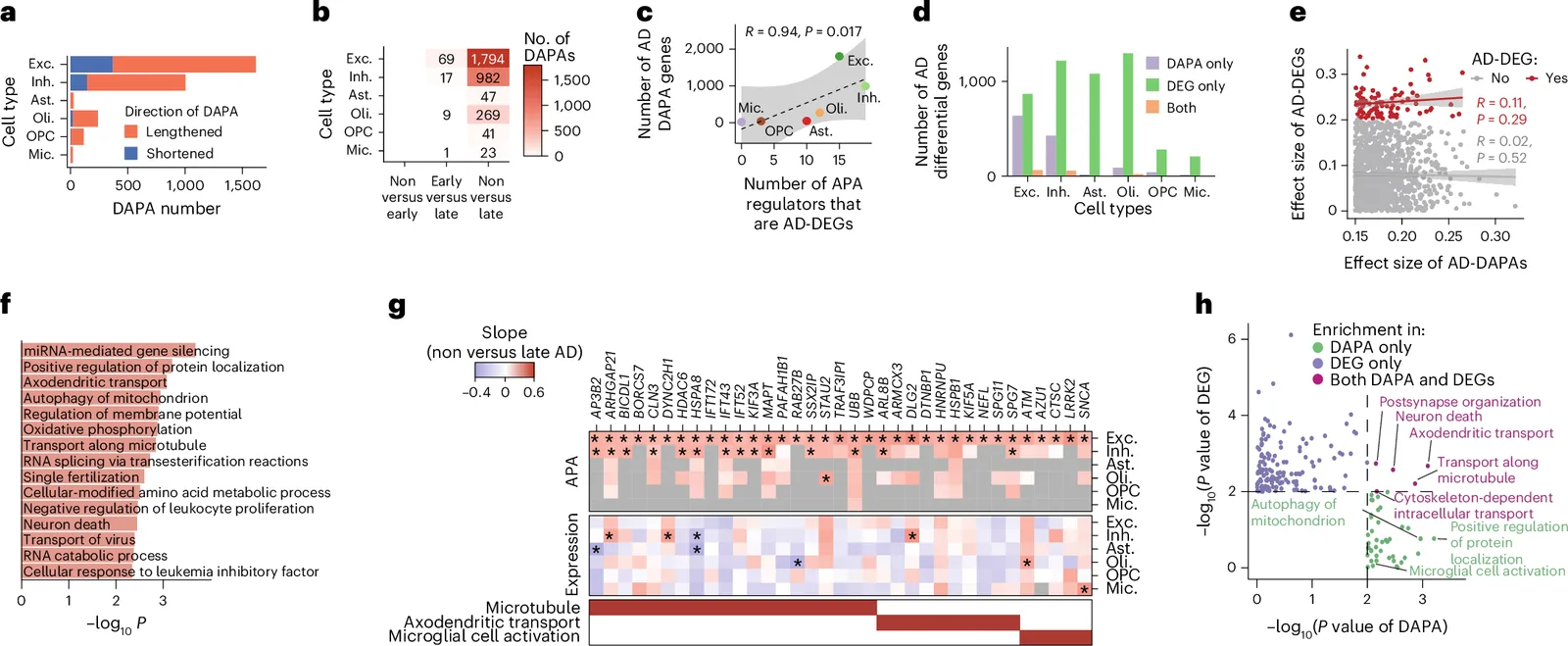
DAPAs in AD. **a**, Numbers of AD-DAPA genes identified in each cell type based on both regression and pairwise analysis between PDUI and AD status, with AD-lengthened and AD-shortened DAPAs represented by different colors. **b**, Numbers of AD-DAPA genes identified in each cell type based on pairwise comparisons among the non-AD, early AD and late AD groups. **c**, Correlations between numbers of AD-DAPAs and numbers of APA regulators that were AD-DEGs across cell types (*P* values and correlation coefficients are from linear regression; ribbon represents the 95% confidence interval). The dashed line represents the predicted mean from the linear regression model. **d**, Intersection of genes that were AD-DAPAs or AD-DEGs in each cell type (for genes tested in both analyses). The longest isoforms of genes were used in the analyses. **e**, Comparison of absolute effect sizes of AD-DAPAs and AD-DEGs. Pearson correlation coefficients and corresponding *P* values were calculated. Only AD-DAPA genes were used, and genes that were also AD-DEGs are plotted in red. Solid lines represent the predicted mean from the linear regression model, and gray shadows denote the 95% confidence intervals of the regression fit. **f**, GO terms enriched for genes with AD-lengthened APAs in excitatory neurons, using genes with APAs in excitatory neurons as the background set. *P* values were calculated using a one-sided hypergeometric test. **g**, Differential APA or expression effects of AD-DAPA genes in key enriched pathways. Heatmaps show per-cell-type AD-DAPA (top) and AD-DEG (center) effect sizes for genes involved in microglial cell activation, axodendritic transport and microtubule pathways (annotations are shown at the bottom). Significant AD-DAPAs or AD-DEGs are indicated by asterisks. **h**, Comparison of enrichment *P* values for GO terms enriched for either AD-lengthened DAPAs or AD-upregulated DEGs in excitatory neurons. *P* values were calculated using a one-sided hypergeometric test.

**Extended Data Fig. 3:**
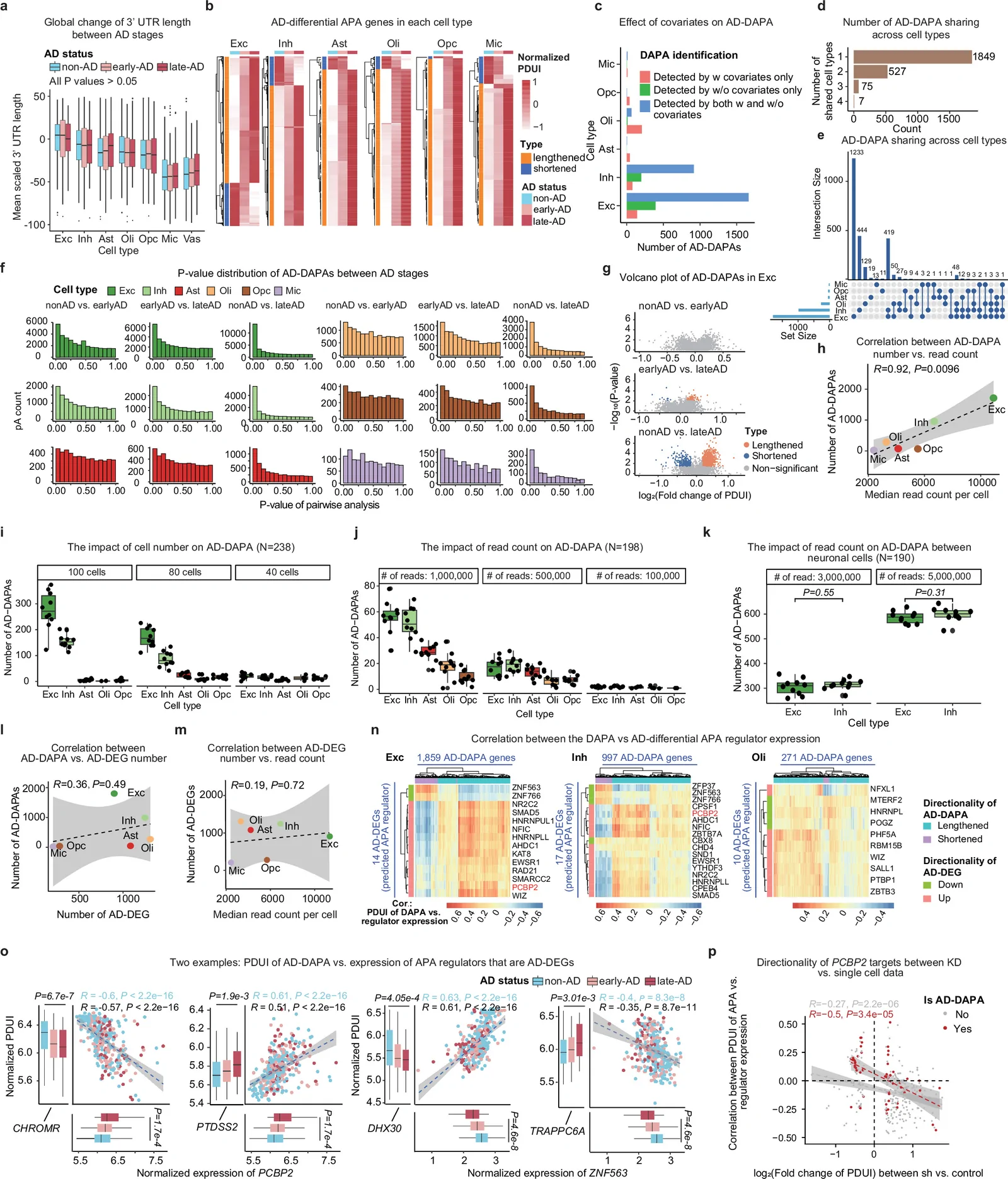
Identification of AD-differential APA across cell types, related to Fig. 2. **a**, Global change of 3’UTR length across cell types during AD progression. P values were calculated by two-sided Wilcoxon tests (N_nonAD_=197, N_earlyAD_ = 121, N_lateAD_ = 61). **b**, Average PDUI of AD-DAPA genes across AD stages and cell types. **c**, Intersection of DAPAs identified with and without sex, age at death and PMI as covariates. **d**, Number of AD-DAPAs shared by different numbers of cell types. **e**, Number of AD-DAPAs shared across cell types. **f**, P-value distribution for pairwise differential APA analyses by two-sided Wilcoxon test. **g**, Volcano plots of pairwise differential APA analyses in excitatory neurons by two-sided Wilcoxon test. **h**, Correlation between AD-DAPA numbers and median read counts per cell across cell types. **i**, AD-DAPA numbers after downsampling (for 10 times) to 100, 80 or 40 cells. **j**, AD-DAPA numbers after downsampling (for 10 times) to 1 M, 500 K, and 100 K reads. **k**, AD-DAPA numbers after downsampling (for 10 times) to 3 M or 5 M reads in excitatory and inhibitory neurons. P values were calculated by two-sided Wilcoxon tests. **l**, Correlation between numbers of AD-DEGs and AD-DAPAs across cell types. **m**, Correlation between AD-DEG number and median reads per cell. **n**, Correlations between PDUI of AD-DAPAs (column) and expression of AD-differential APA regulators (row) across cell types. **o**, Representative examples of the pairs of AD-DAPAs and AD-differentially-expressed regulators in excitatory neurons. For scatter plots, Pearson correlation coefficients and the corresponding P values were calculated. For boxplot, P values were estimated by two-sided Wilcoxon tests. **p**, Fold change between PCBP2 knockdown and control in SH-SY5Y-derived neurons (x-axis) versus the correlation between PDUI of APA and regulator expression (y-axis) in the excitatory neurons of single-cell data. In panels **a, i-k** and **o**, box plots display the medians, upper quartiles and lower quartiles; whiskers extending to 1.5× IQR; outliers are defined as ±1.5× IQR. In panels **h,l,m,o,p**, lines and shading indicate linear regression fits and 95% confidence intervals; Pearson correlations and corresponding P values were calculated.

We next examined APA changes during AD progression. Although we did not observe global shortening or lengthening of 3′ UTRs (Extended Data Fig. 3a), we identified 1,859 and 997 genes in excitatory and inhibitory neurons with DAPAs, respectively (*n* = 23 to *n* = 271 in other cell types) (Fig. 2a, Extended Data Fig. 3b,c, Supplementary Table 7 and Methods). The majority (75.52%) of DAPAs were observed in only one cell type, with shared DAPAs primarily coming from neuron types (Extended Data Fig. 3d,e). In addition, pairwise comparisons between AD stages showed that most changes happened in late AD (Fig. 2b and Extended Data Fig. 3f,g).

We next considered what might determine the different numbers of DAPAs across cell types. Although the number of DAPAs was correlated with sequencing depth (Extended Data Fig. 3h), we found that neuron types had comparable numbers of DAPAs, as well as having more DAPAs than nonneuronal cells in downsampling experiments (Extended Data Fig. 3i–k and Supplementary Note). The number of DAPAs in a cell type was also significantly correlated with the number of APA regulators that were differentially expressed genes (DEGs) in AD (*P* = 0.017, Fig. 2c). By contrast, no correlation was observed between the overall number of DEGs and the number of DAPAs (*P* = 0.49) or sequencing depth (*P* = 0.19, Extended Data Fig. 3l,m).

Expression levels of AD-DAPA regulators were highly correlated with PDUI values of AD-DAPAs (Extended Data Fig. 3n,o), suggesting that differentially expressed AD regulators may modulate AD-DAPAs. In an experiment involving knockdown of *PCBP2*, an AD-DEG and predicted regulator, we identified 789 APA targets of *PCBP2* (61 of which were also AD-DAPAs) (Fig. 1f and Extended Data Fig. 3p). The PDUI values for target APAs were both significantly correlated with the predicted effects in the single-cell data and showed consistent effect directions (all: 71.1%; DAPA: 67.2%), suggesting that changes in *PCBP2* expression may partially contribute to DAPAs during AD progression (Extended Data Fig. 3p).

**Extended Data Fig. 4:**
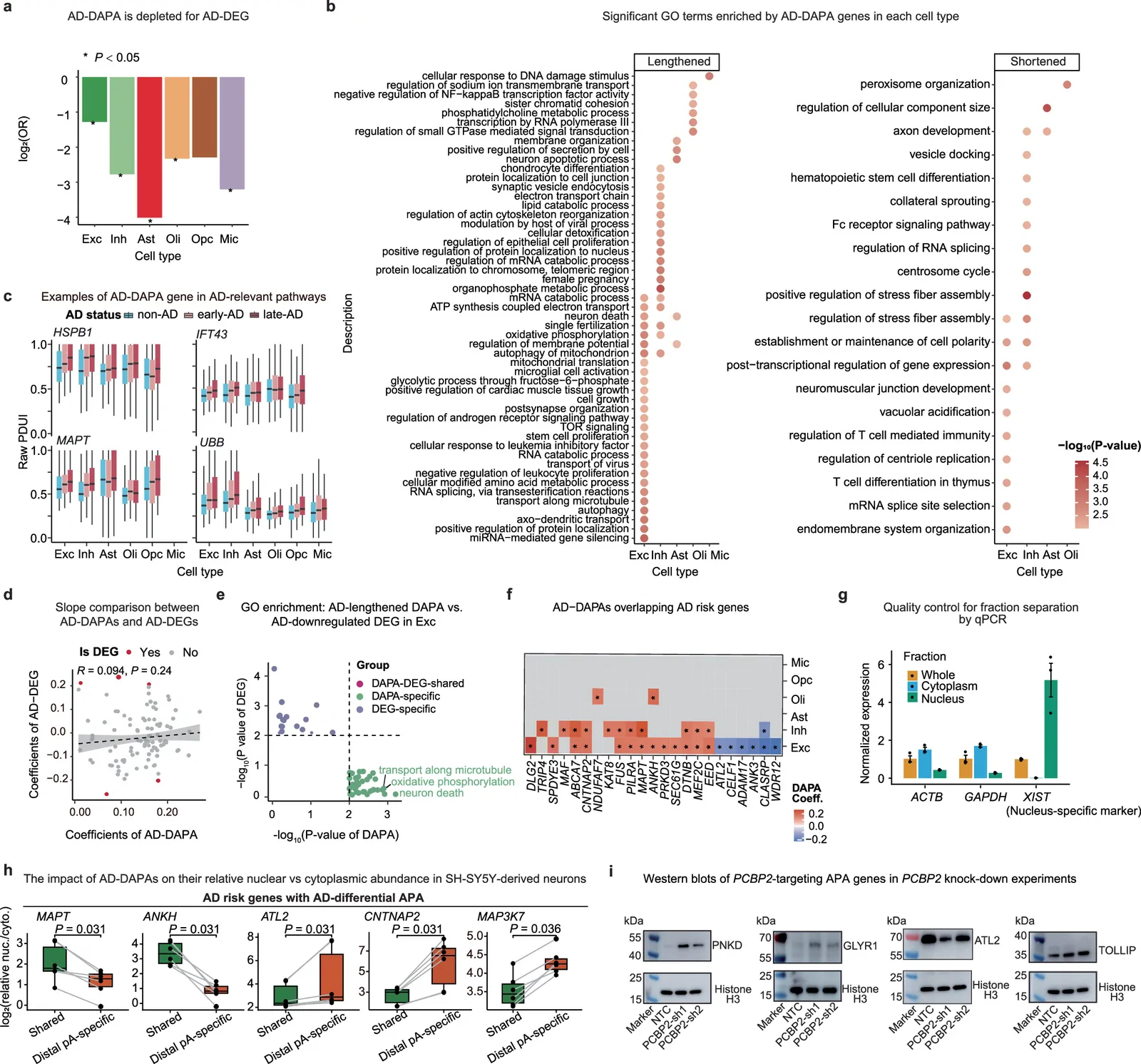
Functional annotation of AD-differential APAs, related to Fig. 2. **a**, Enrichments of AD-DAPA genes against AD-DEGs in each cell type estimated by two-sided Fisher’s test (exact P-values: Exc=1.91×10^−9^, Inh=4.14×10^−41^, Ast=0.00026, Oli=2.36×10^−10^, Opc=0.14, Mic=0.009). **b**, Significant GO terms enriched by AD-lengthened (left) and AD-shortened (right) APA genes across cell types. P value was calculated using a one-sided hypergeometric test. **c**, PDUI comparison between different AD groups for *HSPB1*, *IFT43*, *MAPT* and *UBB* as examples of AD-DAPA genes involved in AD-relevant pathways (N_nonAD_=197, N_earlyAD_ = 121, N_lateAD_ = 61). The box shows the 25th–75th percentile, the line shows the median and the whiskers show 1.5× IQR. **d**, Comparison of the slopes between AD-DAPAs and AD-DEGs for the AD-DAPA genes in the three AD-relevant pathways. The solid line represents the predicted mean from the linear regression model, the gray shadow denotes the 95% confidence intervals of the regression fit. Pearson correlation coefficients and the corresponding P values were calculated. **e**, Comparison of the GO terms enriched by the AD-lengthened DAPAs and AD-downregulated DEGs in the excitatory neurons. P value was calculated using a one-sided hypergeometric test. **f**, Heatmap of AD-DAPA coefficients for AD risk genes with significant AD-DAPA effects. **g**, Relative RNA expression (y-axis) of a nuclear-specific RNA, *XIST*, as well as *ACTB*, and *GAPDH* (x-axis) in different cell fractions upon nucleus-cytoplasm separation experiments (N = 3). The relative expressions in the nuclear and cytoplasmic fractions were quantified by scaling the mean expression in the whole cell fraction to 1. Error bars represent the mean ± standard error. **h**, The relative nuclear cytoplasmic abundance mediated by the 3’UTRs with proximal (green) and distal (red) pA sites for the AD-DAPA genes that were AD risk genes. Statistical tests were calculated using a two-sided paired Wilcoxon test based on 6 replicates for each gene. The box shows the 25th–75th percentile, the line shows the median and the whiskers show 1.5× IQR. **i**, Western blots of PCBP2-targeting APA genes that were also AD-DAPAs in PCBP2-knockdown versus control cells. The PCBP2 knockdown was carried out in SH-SY5Y-derived neurons, using two independent shRNAs. Source data

Notably, genes with DAPAs were significantly depleted for AD-DEGs (5.4–10.7% overlap, odds ratio (OR): 0.006–0.410) (Fig. 2d, Extended Data Fig. 4a and Supplementary Table 8). In addition, we found no correlation between the absolute effect sizes of AD-DAPAs and AD-DEGs (Fig. 2e), suggesting that they were largely orthogonal. AD-lengthened genes in neurons were enriched for multiple processes that have been implicated in AD etiology and progression, including microglial cell activation, microtubule transport, axodendritic transport and mitochondria-related pathways^48–51^, and involve AD-implicated genes such as *MAPT* and *UBB*^52,53^ (Fig. 2f,g, Extended Data Fig. 4b,c and Supplementary Table 9). Most genes in these pathways were not AD-DEGs, and there was no correlation between their DAPA and DEG effect sizes (*R* = 0.094, *P* = 0.24) (Extended Data Fig. 4d). Whereas the microglial activation and mitochondrial pathways were AD-DAPA-specific, both AD-DAPAs and AD-DEGs for excitatory neurons were enriched for microtubule and axodendritic transport (Fig. 2h, Extended Data Fig. 4e and Supplementary Table 10). Overall, these results indicate that DAPAs represent an independent set of genes that are dysregulated during AD progression and partly converge on the same disease pathways with expression changes.

We next investigated the downstream functional consequences of DAPAs for AD risk genes. We found that 23 of 180 putative AD risk genes were AD-DAPAs; these included key GWAS locus genes *MAPT*, *ABCA7* and *MEF2C* and rare-variant genes *DLG2* and *DTNB*^54,55^ (Extended Data Fig. 4f). We selected five AD risk genes and determined whether their DAPAs changed the relative abundance of their transcripts in nuclei versus cytoplasm of SH-SY5Y-derived neurons (Extended Data Fig. 4g,h and Supplementary Note). For all genes, proximal and distal 3′ UTRs showed significant differences in abundance between cellular fractions, probably owing to differential effects of 3′ UTRs on mRNA degradation or localization^56^ (Extended Data Fig. 4h). Finally, we explored whether dysregulation of APA in AD could affect protein abundance. Four of five tested AD-DAPA targets of *PCBP2* showed clear changes in protein levels in SH-SY5Y *PCBP2* knockdown experiments (Extended Data Fig. 4i), with three increasing and one decreasing in abundance, consistent with findings that the effects of APAs on translation and mRNA stability may vary across different genes^1,21,57^. Overall, these results suggest that APA regulation can contribute to AD by altering cellular localization and protein abundance through its effects on mRNA stability, localization and translation.

### Genetic regulation of APAs in brain cell types

**Fig. 3:**
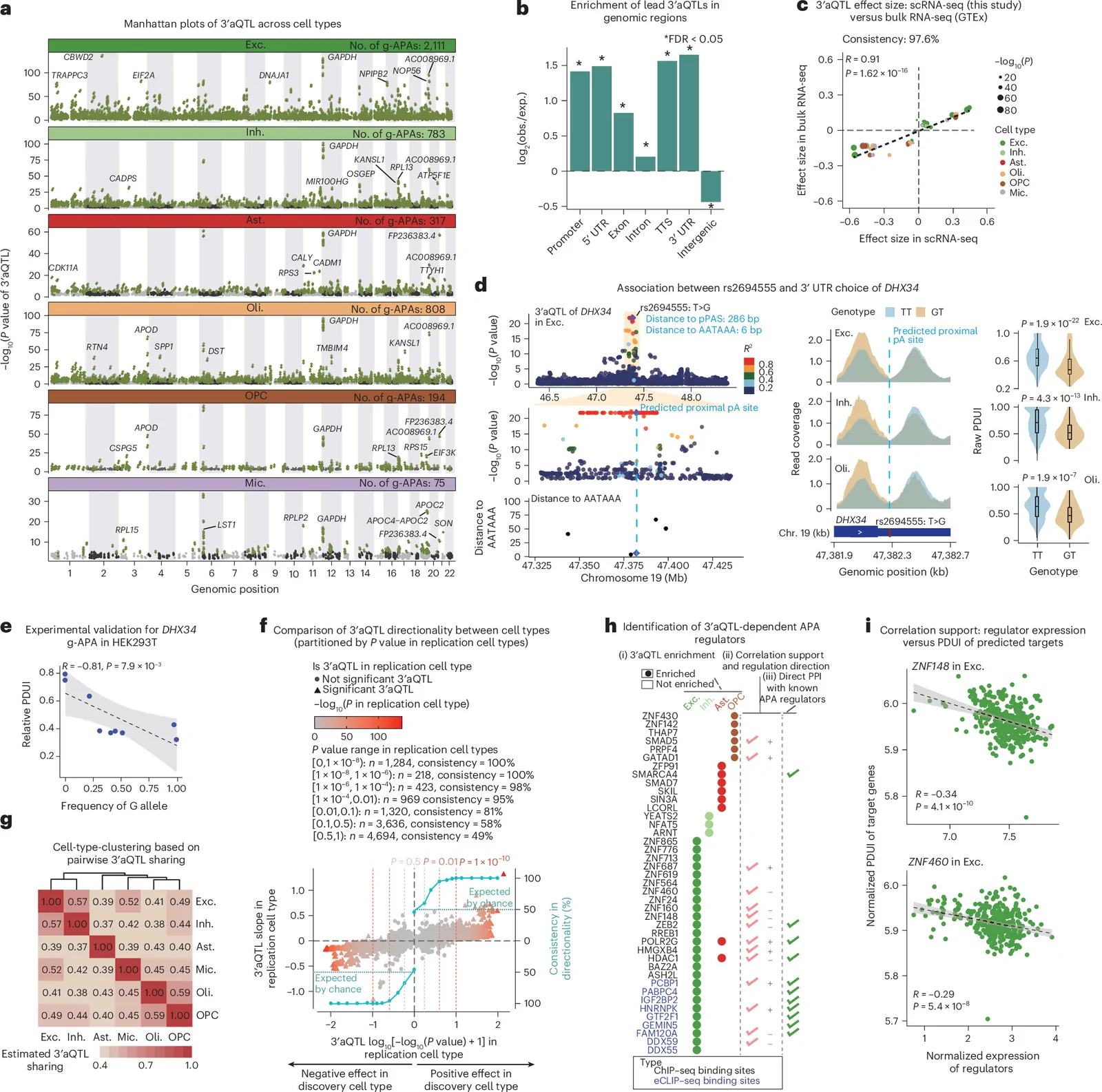
Cell-type-resolved 3′aQTLs in the brain. **a**, Manhattan plot showing nominal *P* values of 3′aQTLs in each cell type. *P* values were calculated by FastQTL linear regression. The top eight 3′aQTLs in each cell type are labeled with the gene names of the corresponding g-APAs. **b**, Enrichments of lead 3′aQTLs for different functional genomic annotations. **c**, Correlations of effect sizes between 3′aQTLs identified at cell type level (*x* axis, from this study) and at the bulk level (*y* axis, from GTEx). The consistency ratio is the percentage of QTLs with consistent directionality between both studies. Pearson correlation coefficients and corresponding *P* values were calculated. **d**, The APA of *DHX34* as an example of a 3′aQTL in three cell types. Left: nominal *P* values of 3′aQTLs surrounding the predicted proximal pA sites (top), with zoomed-in view of the distance to the closest AATAAA motif for each variant (bottom). LD correlations between the lead variant and other variants are indicated by color. Middle: raw coverage of reads with different genotypes at the rs2694555 position. Right: boxplot of PDUIs at the individual level for each genotype (*n*_TT_ = 316, *n*_GT_ = 39). The box shows the 25th–75th percentile, the line shows the median and whiskers show 1.5× IQR. *P* values were calculated by FastQTL linear regression. **e**, Prime-editing-based validation of the 3′aQTL for the *DHX34* APA. Scatter plot showing the correlation between frequency of the G allele of different edited lines (*x* axis) versus the relative PDUI of *DHX34* estimated based on quantitative PCR experiments (*y* axis). Correlations and *P* values were calculated based on nine edited HEK293T lines using two-sided Pearson’s correlation test. The dashed line represents the predicted mean from the linear regression model; gray shadow denotes the 95% confidence intervals of the regression fit. **f**, Consistency in the directionality (right *y* axis) of 3′aQTL effects (left *y* axis) between one cell type and a replication cell type, plotted against the *P* value of the 3′aQTL in the replication cell type times the sign of the 3′aQTL in the discovery cell type (*x* axis). *P* values were calculated by FastQTL linear regression. Top: consistency in directionality in replication cell types for 3′aQTLs with different significance cutoffs. **g**, Heatmap of the estimated fraction of shared 3′aQTLs between pairs of cell types. **h**, Predicted 3′aQTL-dependent regulators for four major cell types. Lines of evidence include (i) enrichment of ChIP–seq or eCLIP–seq binding sites for 3′aQTLs in each cell type; (ii) significant correlations between the PDUIs of the regulator and its targets, with both positive (purple) and negative (blue) correlations shown; (iii) direct protein–protein interactions with one or more known APA regulators. **i**, Correlations between excitatory neuron gene expression (*x* axis) of *ZNF148* (top) and *ZNF460* (bottom) and the normalized PDUI of their target genes (*y* axis). *P* values and correlation coefficients were from linear regression; the ribbon indicates the 95% confidence interval. The dashed line represents the predicted mean from the linear regression model. *FDR < 0.05. exp., expected; obs., observed; PPI, protein–protein interaction; scRNA-seq, single-cell RNA-seq; TTS, transcriptional terminal site.

**Extended Data Fig. 5:**
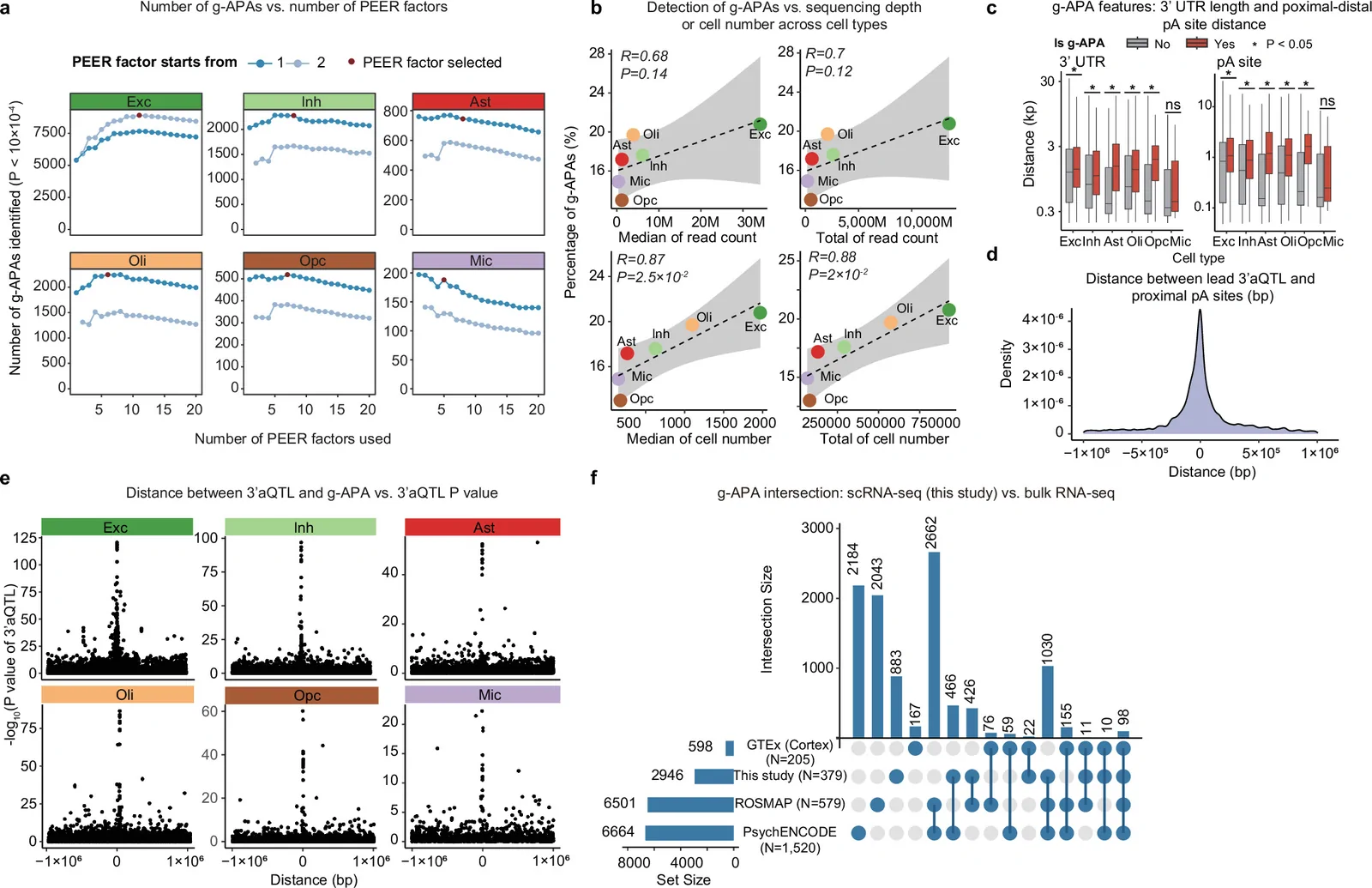
3’aQTL landscape in each cell type, related to Fig. 3. **a**, Number of g-APAs (nominal P < 10^−4^) obtained when using different PEER factors for 3’aQTL identification (a parameter testing). The PEER factor used for the 3’aQTL identification was denoted by a red dot for each cell type. **b**, Cross-cell-type correlation between the number of g-APAs identified versus cell number or sequencing depth. Solid lines represent the predicted mean from the linear regression model, and gray shadows denote the 95% confidence intervals of the regression fit. Pearson correlation coefficients and the corresponding P values were calculated. **c**, Comparison of 3’UTR length (left) and proximal-to-distal pA sites (right) between g-APAs and the APAs without significant 3’aQTLs, where P values were estimated by two-sided Wilcoxon tests (exact P-values of pA site-length: Exc=3.24×10^−11^, Inh=2.54×10^−4^, Ast=1.36×10^−12^, Oli=2.08×10^−9^, Opc=1.4×10^−8^, Mic=0.11; exact P-values of 3’UTR length: Exc=5.0×10^−7^, Inh=0.0017, Ast=3.07×10^−11^, Oli=1.01×10^−7^, Opc=8.28×10^−9^, Mic=0.14; Exc: N_Yes_ = 629 and N_No_ = 4,441; Inh: N_Yes_ = 160 and N_No_ = 1,967; Ast: N_Yes_ = 89 and N_No_ = 849; Oli: N_Yes_ = 200 and N_No_ = 1,780; Opc: N_Yes_ = 54 and N_No_ = 690; Mic: N_Yes_ = 22 and N_No_ = 213). The box shows the 25th–75th percentile, the line shows the median and the whiskers show 1.5× IQR. **d**, Distribution of the distance between lead 3’aQTLs and the corresponding proximal pA sites. **e**, Distance between 3’aQTLs to the associated APAs (x-axis) versus the nominal P-values of 3’aQTLs (y-axis) in each cell type. P-values were calculated by FastQTL linear regression. **f**, Upset plot showing the intersection between the g-APAs identified at the cell type level (by this study) and at the bulk level (by GTEx, ROSMAP and PsychENCODE), with the g-APAs from all the cell types merged and sample size annotated. All g-APAs were de-duplicated at gene level.

We next called *cis* 3′aQTLs and identified 4,288 genes with g-APAs (*q*-value < 0.05) after accounting for multiple testing and nongenetic factors (Fig. 3a, Extended Data Fig. 5a, Supplementary Table 11 and Methods). We identified the most g-APAs in excitatory neurons (2,111) and the fewest in microglia (75) (Fig. 3a), possibly owing to differences in cell numbers and sequencing depth (Extended Data Fig. 5b). Compared to other APAs, g-APAs had longer 3′ UTRs with proximal and distal pA sites further apart (Extended Data Fig. 5c). Lead 3′aQTLs were preferentially located near their g-APAs, and g-APAs near 3′aQTLs were more significant (Extended Data Fig. 5d,e). Lead 3′aQTLs were enriched in 3′ UTRs (OR = 3.14) but also in promoters and 5′ UTRs (ORs: 2.67 and 2.81), consistent with recent observations that promoters can affect APA^1,58^ (Fig. 3b).

We identified 883 loci not found in other cohorts, and the remainder of our 3′aQTLs strongly overlapped with GTEx, ROSMAP and PsychENCODE 3′aQTLs^25,26^ (Extended Data Fig. 5f). Comparison with the GTEx brain cortex 3′aQTLs, for which effect sizes were available, showed highly consistent effects, with 97.6% concordant directions and strongly correlated effect sizes (*R* = 0.91)^25^ (Fig. 3c and Methods).

**Extended Data Fig. 6:**
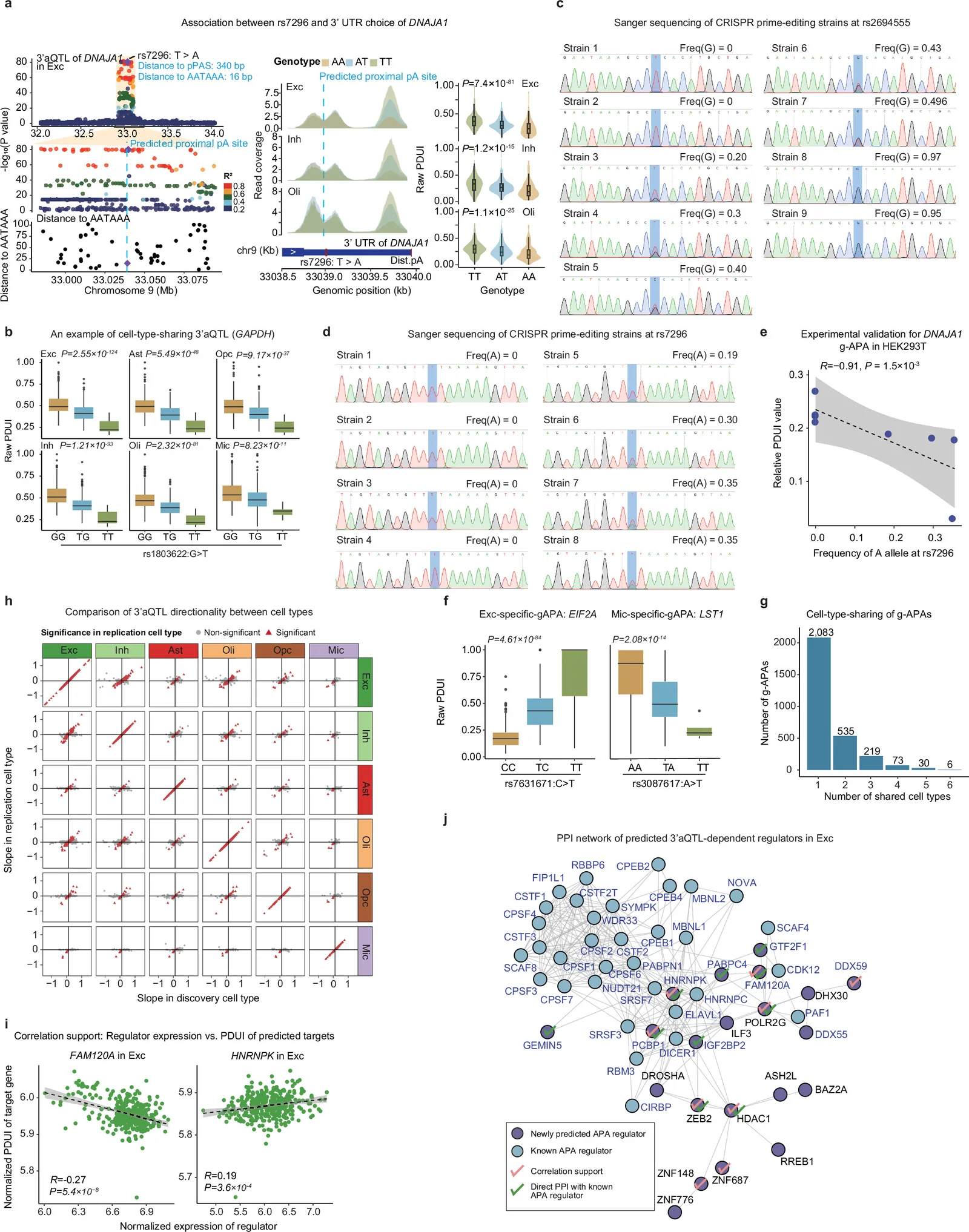
Cell type specificity of 3’aQTLs, related to Fig. 3. **a**, *DNAJA1* APA as an example of a 3’aQTL in three cell types. Left: Nominal P-values from FastQTL linear regression (top), with the zoom-in view for distance to the closest AATAAA motif (bottom). Colors indicate LD with the lead variant. Middle: read coverage with different rs7296 genotypes. Right: PDUI by genotypes (N_AA_ = 51, N_AT_ = 153 and N_TT_ = 144). **b**, *GAPDH* APA as an example of cell-type-sharing 3’aQTLs (N_GG_ = 206, N_TG_ = 112 and N_TT_ = 15). P values were calculated by FastQTL linear regression. The box shows the 25th–75th percentile, the line shows the median and the whiskers show 1.5× IQR. **c,d**, Sanger sequencing of CRISPR prime-edited lines for rs2694555 in DHX34 (**c**) and rs7296 in DNAJA1 (**d**). Target variants are highlighted in blue, with estimated genotype frequencies indicated. **e**, Prime-editing validation of the DNAJA1 3′aQTL. A-allele frequency across eight edited HEK293T lines was correlated with relative DNAJA1 PDUI measured by qPCR (two-sided Pearson correlation). Lines and shading indicate linear regression fits and 95% confidence intervals. **f**, *EIF2A* in excitatory neurons (N_CC_ = 239, N_TC_ = 84 and N_TT_ = 10) and *LST1* in microglia (N_AA_ = 186, N_TA_ = 27 and N_TT_ = 4) as cell-type-specific 3’aQTL examples, respectively. P-values were calculated by FastQTL linear regression. The box shows the 25th–75th percentile, the line shows the median and the whiskers show 1.5× IQR. **g**, Number of the g-APAs (y-axis) shared by different numbers of cell types (x-axis). **h**, Pairwise comparisons of 3’aQTL effect sizes between different cell types. The 3’aQTLs that were significant in the replication cell types (y-axis) were labeled by red triangles. **i**, Correlation between the gene expression of predicted regulators (x axis) and the normalized PDUI of their target genes (y-axis) for *FAM120A* and *HNRNPK* as examples of regulators identified in excitatory neurons. Lines and shading indicate linear regression fits and 95% confidence intervals. Pearson correlation coefficients and the corresponding P values were calculated. **j**, PPI network formed by the newly predicted 3’aQTL-dependent APA regulators and the known APA regulators.

Several 3′aQTLs were shared across multiple cell types, including g-APAs for *GAPDH* across all six cell types; *DHX34* and *DNAJA1* in excitatory neurons, inhibitory neurons and oligodendrocytes; and *APOD* in oligodendrocytes and OPCs^59^ (Fig. 3a,d and Extended Data Fig. 6a,b). We experimentally validated two loci, rs2694555 for *DHX34* (also an AD-DAPA gene) and rs7296 for *DNAJA1*, using CRISPR-based prime editing in HEK293T cells (Supplementary Note). Editing level was strongly correlated with PDUIs in each target (Pearson correlations: 0.81 and 0.91, respectively) (Fig. 3e and Extended Data Fig. 6c–e).

Overall, 2,083 g-APA loci (70.7%) were found in only one cell type, such as *EIF2A* in excitatory neurons and *LST1* in microglia (Extended Data Fig. 6f,g and Supplementary Table 12). As 3′aQTL discovery was influenced by cell number (Extended Data Fig. 5b), we estimated QTL sharing based on effect-direction concordance, which is relatively less affected by power differences^60,61^. Significant 3′aQTLs shared by cell types were 100% concordant, and 3′aQTLs were 95% consistent down to *P* = 10^−2^ (Fig. 3f, Extended Data Fig. 6h and Methods). We estimated the fraction of cell-type-sharing 3′aQTLs to be 37–59%, with the highest levels between neuron types (57%) and between oligodendrocytes and OPCs (59%) (Fig. 3g).

To infer regulators underlying g-APAs, we searched for both RBPs and TFs with binding sites that were enriched near 3′aQTLs^25^. We integrated 3′aQTLs with data from enhanced cross-linking and immunoprecipitation followed by sequencing (eCLIP–seq; for RBPs) and chromatin immunoprecipitation followed by sequencing (ChIP–seq; for TFs) and found 9 RBPs and 32 TFs with binding sites enriched near 3′aQTLs in at least one cell type^62^ (Fig. 3h, Supplementary Table 13 and Methods). Two-thirds of the RBPs were also significantly enriched near GTEx bulk 3′aQTLs. A subset of four regulators had expression levels that were significantly correlated with the average PDUIs of their target genes (Fig. 3h,i, Extended Data Fig. 6i and Supplementary Table 13). These included RBPs FAM120A and HNRNPK (Extended Data Fig. 6i), and TFs ZNF148 and ZNF460, all of which have been implicated in brain diseases^42,63–65^ (Fig. 3i). These regulators formed a tight protein–protein interaction network that included known and prioritized APA regulators (Extended Data Fig. 6j and Supplementary Table 13), suggesting a potential cooperative regulatory mode for APA.

### 3′aQTLs preferentially colocalize with pQTLs over eQTLs

**Fig. 4:**
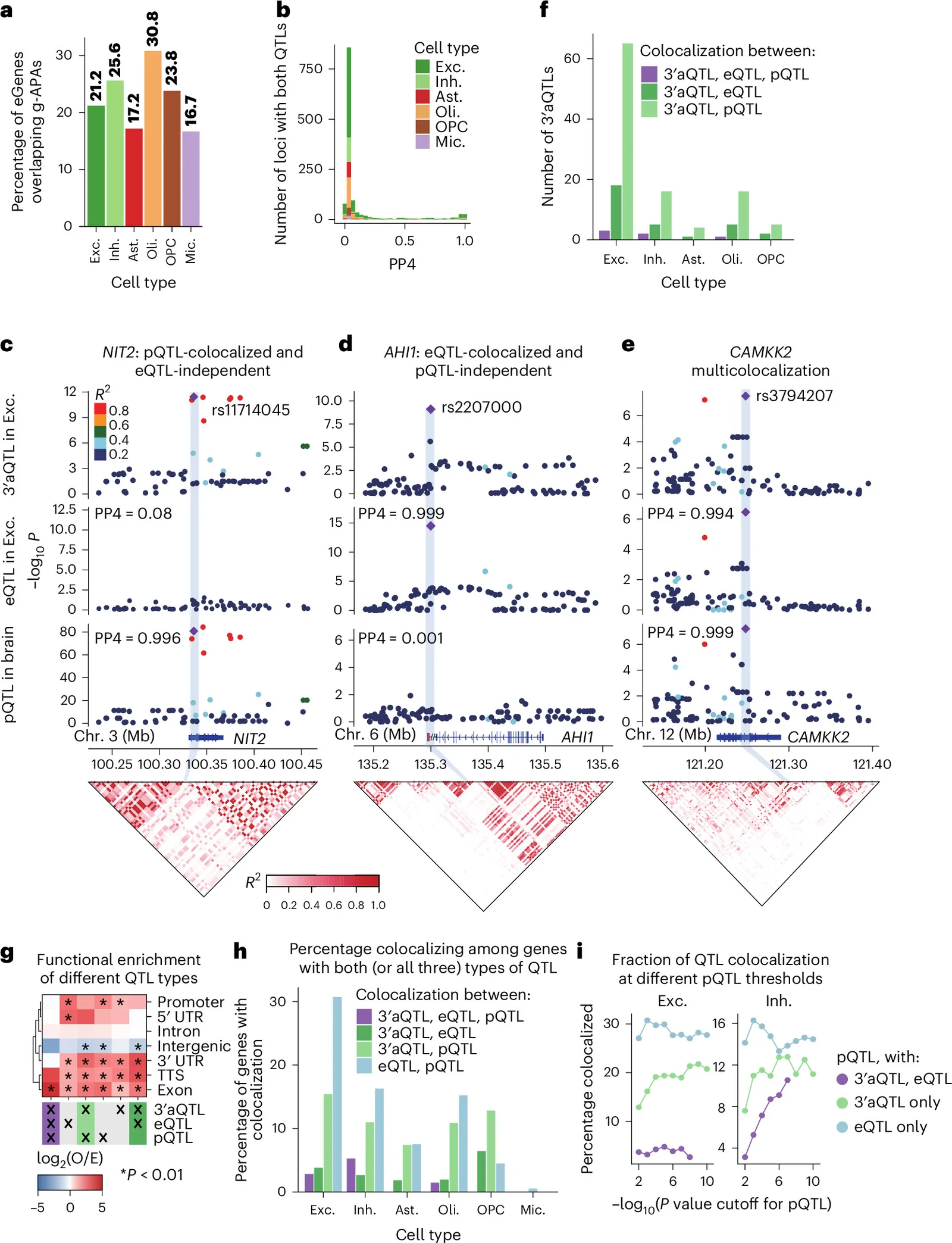
Integrative analysis between 3′aQTLs, eQTLs and pQTLs. **a**, Percentage overlap between eGenes and g-APA genes in each cell type. **b**, Distribution of colocalization posterior probability between 3′aQTLs and eQTLs for the genes that were both eGenes and g-APA genes. 3′aQTLs were largely orthogonal to eQTLs. **c**, The *NIT2* locus in excitatory neurons as an example of eQTL-independent colocalization between 3′aQTLs and pQTLs. Nominal *P* values of 3′aQTLs, eQTLs and pQTLs are shown. *P* values were calculated by FastQTL linear regression. **d**, The *AHI1* locus in excitatory neurons as an example of pQTL-independent colocalization between 3′aQTLs and eQTLs. *P* values were calculated by FastQTL linear regression. **e**, The *CAMKK2* locus in excitatory neurons as an example of multicolocalization across 3′aQTLs, eQTLs and pQTLs. *P* values were calculated by FastQTL linear regression. **f**, Numbers of different types of colocalization between 3′aQTLs versus eQTLs and pQTLs in each cell type. For each colocalization analysis group, only genes with significant QTLs in both molecular traits were used. **g**, Enrichments of 3′aQTLs with different colocalization modes for different genomic annotations, estimated using Fisher’s exact test. Significant enrichments (red) or depletions (blue) are labeled with asterisks. **h**, Fractions of colocalization for different modes of colocalization between 3′aQTLs, eQTLs and pQTLs in each cell type. Proportions were calculated as the number of colocalized loci divided by the number of loci used for the colocalization analysis. **i**, Fractions of pQTLs colocalized with eQTLs (in blue), 3′aQTLs (in green) or both (in purple) when different thresholds were used to select significant pQTLs for the colocalization analysis. Proportions were calculated as the number of colocalized loci divided by the number of loci used for the colocalization analysis. O/E, fold change of observed versus expected values.

**Extended Data Fig. 7:**
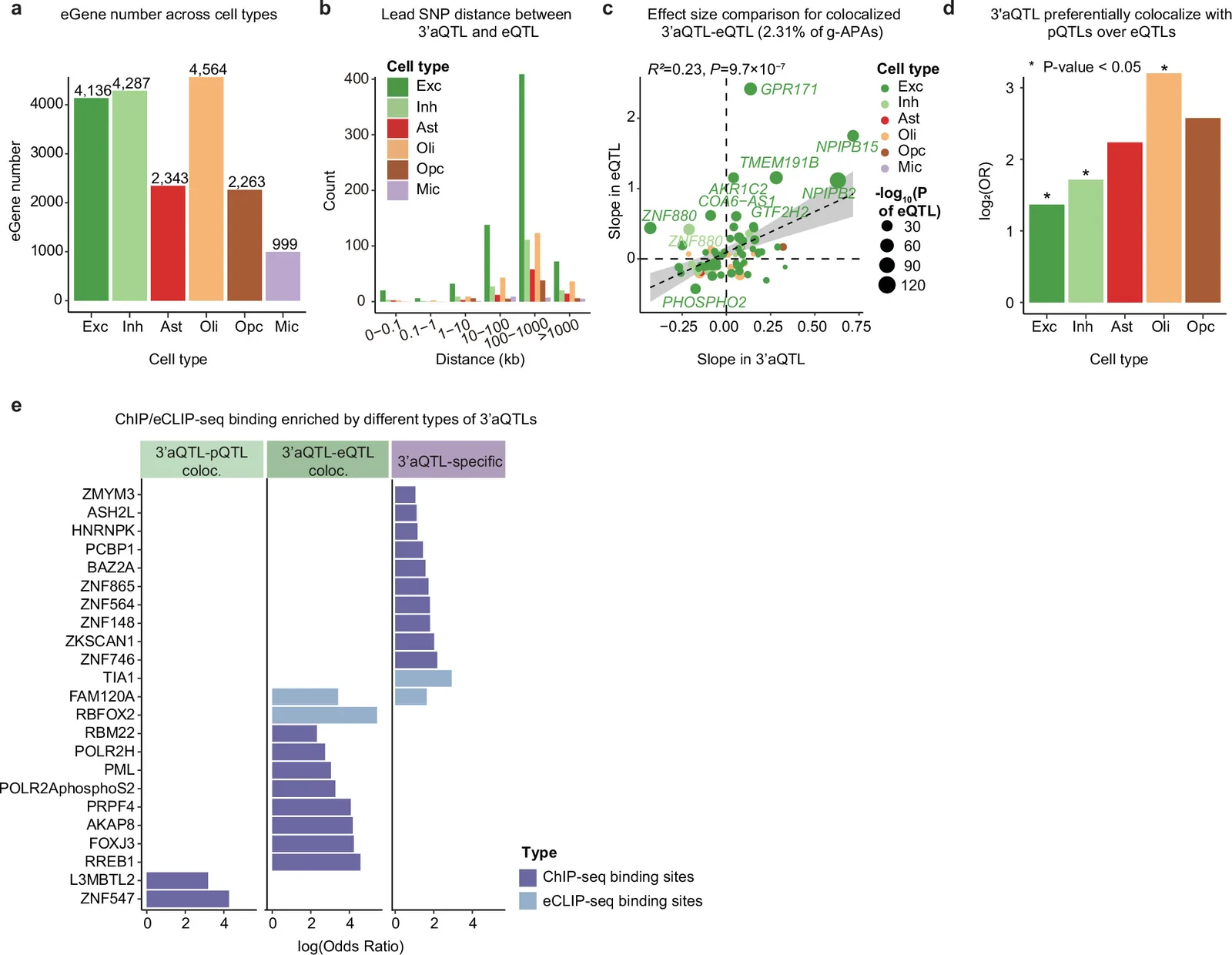
Colocalization analysis between 3’aQTLs, eQTLs and pQTLs, related to Fig. 4. **a**, Number of eGenes identified for each cell type using the same snRNA-seq dataset. **b**, Number of loci (y-axis) within different ranges of distance (x-axis) between the lead 3’aQTLs and lead eQTLs across cell types. **c**, Correlation of effect sizes between 3’aQTLs (x-axis) and eQTLs (y-axis) for the colocalized 3’aQTL-eQTL pairs. Pearson correlation coefficients and the corresponding P values were calculated. The solid line represents the predicted mean from the linear regression model, and the gray shadow denotes the 95% confidence intervals of the regression fit. **d**, Relative enrichments of the 3’aQTL-pQTL colocalized loci over the 3’aQTL-eQTL colocalized genes in each cell type, where log_2_(OR) > 0 indicating that 3’aQTL preferentially colocalized with pQTL over eQTL. OR and enrichment significance were calculated by a two-sided Fisher’s exact test (exact P-values: Exc=6.88×10^−5^, Inh=0.013, Ast=0.34, Oli=7.04×10^−6^, Opc=0.07). **e**, Enrichments of ChIP-seq (in purple) or eCLIP-seq (in blue) binding sites in the loci with different colocalization modes between 3’aQTL versus eQTLs and pQTLs.

As 3′UTR selection can affect mRNA levels by regulating mRNA stability, we next compared 3′aQTLs with eQTLs that we had identified from the same snRNA-seq dataset^5^ (Extended Data Fig. 7a, Supplementary Table 14 and Methods). Across cell types, we observed an overlap of 16.7–30.8% in the set of genes with eQTLs and 3′aQTLs (Fig. 4a). However, 73.1% of the lead 3′aQTL and eQTL variants of these loci were more than 100 kb apart, and 94.3% of the loci had low colocalization posterior probability (PP4 < 0.1) (Fig. 4b and Extended Data Fig. 7b). For colocalized loci (2.3%), we observed a modest positive correlation between expression and APA QTL effect sizes (Extended Data Fig. 7c and Methods). Overall, our results suggest that 3′aQTLs are largely orthogonal to eQTLs.

Given the role of 3′ UTRs in regulation of both translation and mRNA stability, we next performed colocalization across 3′aQTLs, eQTLs and published bulk brain pQTLs^28,66,67^ (Fig. 4c–f, Supplementary Table 15 and Methods). We identified multiple types of locus: 3′aQTL–pQTL (*n* = 102), 3′aQTL–eQTL (*n* = 31) and 3′aQTL–eQTL–pQTL multicolocalization (*n* = 6) (Fig. 4c–f). The 3′aQTL–pQTLs colocalized more often than 3′aQTL–eQTLs across cell types (OR: 2.58–9.20) (Extended Data Fig. 7d). These different modes showed distinct genomic enrichments: 3′aQTL–eQTL colocalized loci were more enriched in transcriptional terminal site regions and 3′ UTRs, whereas 3′aQTL–pQTLs were more enriched in 3′ UTRs and exons than 3′aQTLs alone (Fig. 4g). In addition, we identified 12 TFs and RBPs with increased binding in 3′aQTL–pQTLs and 3′aQTL–eQTLs, including two RNA polymerase II subunits, which were enriched for 3′aQTL–eQTLs and could be involved in a potential transcription-coupled mechanism underlying pA site selection (Extended Data Fig. 7e).

We next quantified the relative prevalence of each QTL colocalization mode. Genes with both 3′aQTLs and pQTLs showed higher colocalization between these QTLs (up to 16.1% of genes) than genes with 3′aQTLs and eQTLs (up to 6.5%) or genes with all three QTLs (up to 5.3%) (Fig. 4h). Although eQTL–pQTL colocalization was most frequent (up to 30% in excitatory neurons), 3′aQTL–pQTL colocalization was comparable at more stringent pQTL significance thresholds, suggesting that our estimates might have been underpowered (Fig. 4h–i). Overall, our results suggest that 3′aQTLs are more tightly coupled to pQTLs than to eQTLs and offer another layer for interpretation of pQTLs in addition to capture of mRNA expression levels.

### Identification of 3′aQTL-dependent AD GWAS loci

**Fig. 5:**
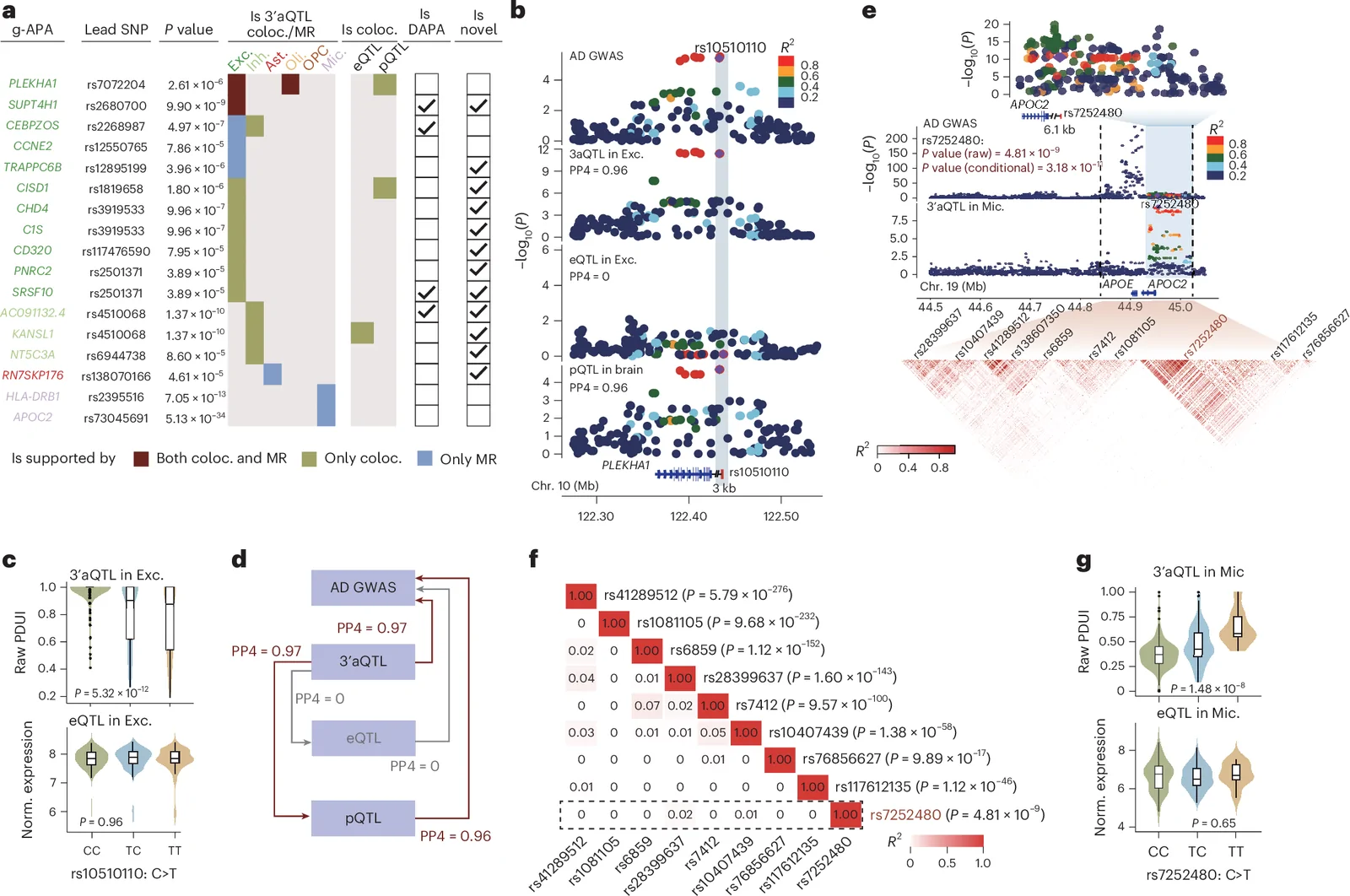
Interpretation of AD GWAS using 3′aQTLs. **a**, Colocalization and MR analysis between cell-type-resolved 3′aQTLs and AD GWAS loci. Columns indicate whether genes had previously been linked to AD through a molecular QTL (‘is novel’); whether loci were also colocalized with eQTLs or pQTLs (‘is coloc.’); and whether APA are differential in AD (‘is DAPA’). **b**, The *PLEKHA1* locus as an example of a 3′aQTL-colocalized AD GWAS risk locus. The locus was also colocalized with pQTLs but not with eQTLs (pQTL-dependent and eQTL-independent). **c**, Boxplots showing per-genotype PDUI (top) and mRNA expression values (bottom) for *PLEKHA1* at rs10510110 in excitatory neurons (*n*_CC_ = 41, *n*_TC_ = 168, *n*_TT_ = 136). The AD-GWAS-colocalized 3′aQTL at the *PLEKHA1* locus was eQTL-independent. The box shows the 25th–75th percentile, the line shows the median and whiskers show 1.5× IQR. **d**, Schematic of the colocalization modes across the AD GWAS locus and each of the three QTL types at the *PLEKHA1* locus in excitatory neurons, showing the posterior probabilities for pairwise colocalization analyses. **e**, Conditional analysis of the *APOC2* AD locus and its 3′aQTL colocalization. AD GWAS and 3′aQTL nominal *P* values at the *APOE* and *APOC2* loci are shown. The inset shows a zoomed-in view of the AD GWAS signal at the *APOC2* locus. Bottom: LD heatmap of the locus, labeled with the SNPs used for the conditional analysis. The lead 3′aQTL SNP for the *APOC2* locus (rs7252480) is highlighted in red. **f**, LD correlations (*R*^2^) between the lead 3′aQTL SNP (rs7252480) and the SNPs used for conditional analysis at the *APOE*/*APOC2* locus; each SNP is labeled with its AD GWAS *P* value. **g**, Boxplots showing the per-genotype PDUI (top) and mRNA expression values (bottom) of *APOC2* for rs7252480 in microglia (*n*_CC_ = 153, *n*_TC_ = 116, *n*_TT_ = 17). AD-GWAS-colocalized 3′aQTL at the *APOC2* locus was eQTL-independent. The box shows the 25th–75th percentile, the line shows the median and whiskers show 1.5× IQR. In **b**, **c** and **e**–**g**, QTL *P* values were calculated by FastQTL linear regression.

**Extended Data Fig. 8:**
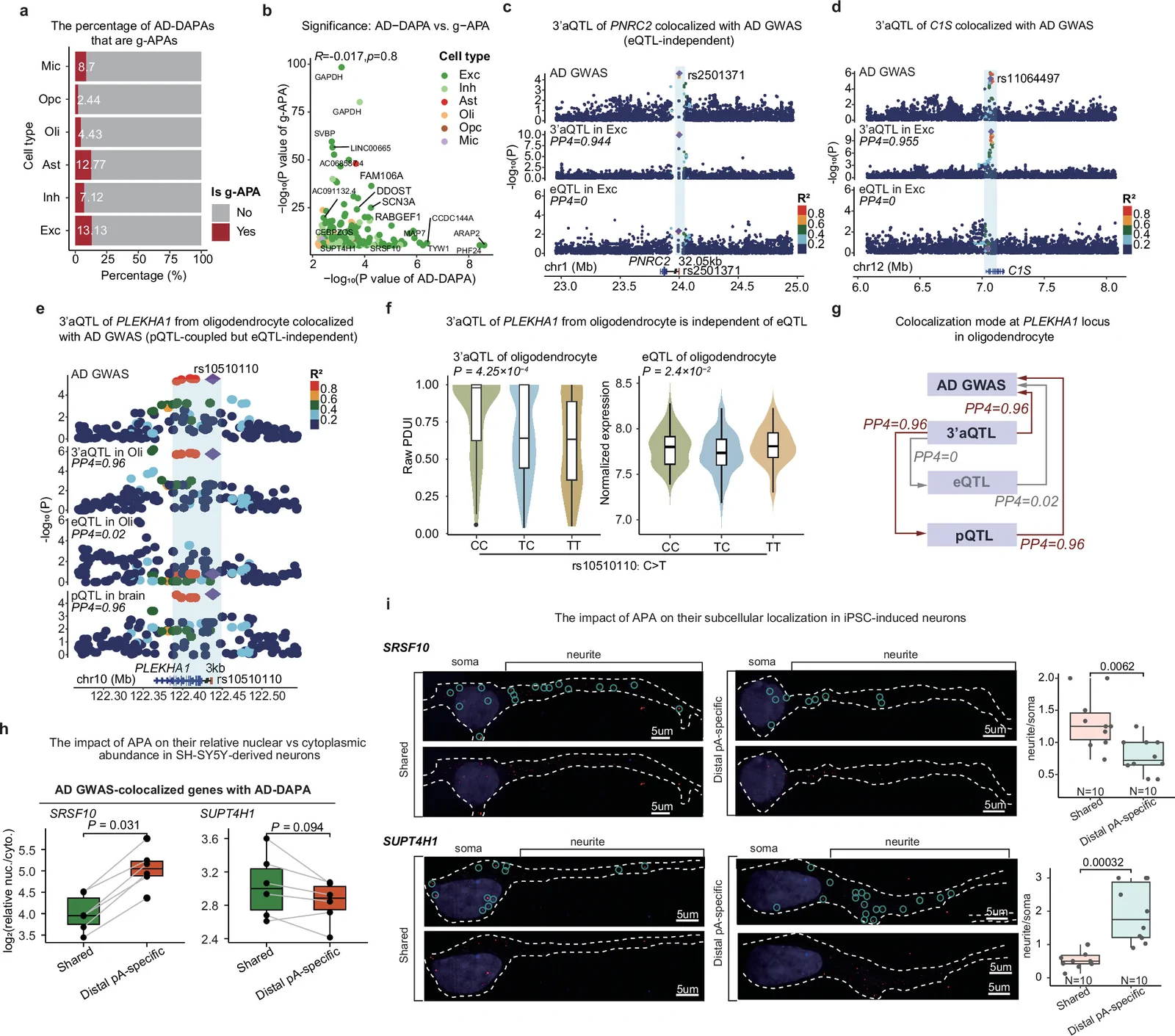
Colocalization between 3’aQTLs and AD GWAS, related to Fig. 5. **a**, Fraction of AD-DAPA genes that are also regulated by 3’aQTLs in each cell type. **b**, Comparison of -log_10_(P-values) for the intersected APAs that are both AD-DAPAs (x-axis) and g-APAs with significant 3’aQTLs (y-axis). Points are colored by major cell type. Top g-APAs, top AD-DAPAs, and the AD GWAS-colocalized loci are labeled. Pearson correlation coefficients and the corresponding P values were calculated. **c-d**, Locus-compare visualization of *PNRC2* (**c**) and *C1S* (**d**) loci as examples of eQTL-independent colocalization between 3’aQTLs and AD GWAS in excitatory neurons. The nominal P-values of AD GWAS (top), 3’aQTLs (middle), and eQTL (bottom) were shown with different panels. **e**, Locus-compare visualization of *PLEKHA1* locus as examples of eQTL-independent and pQTL-dependent colocalization between 3’aQTLs and AD GWAS in oligodendrocyte. **f**, Boxplots showing the PDUI (left) and mRNA expression (right) of *PLEKHA1* with different genotypes at rs10510110 locus in oligodendrocyte (N_CC_ = 36, N_TC_ = 148 and N_TT_ = 123). **g**, The colocalization mode across AD GWAS, 3’aQTL, eQTL and pQTL at the *PLEKHA1* locus in oligodendrocyte. **h**, The relative proportion of mRNA abundance in nucleus versus cytoplasm mediated by the 3’UTRs with proximal (green) and distal (red) pA sites for two AD-GWAS-colocalized genes. Statistical tests were calculated using two-sided paired Wilcoxon tests, based on 6 replicates for each gene. **i**, smiFISH experiments showing the soma vs neurite localization of *SRSF10* (upper) and *SUPT4H1* (lower) in iPSC-derived neurons. The left panels show representative cells with probes targeting long-isoform-specific and shared regions. The right panels show the quantified neurite/soma ratios. P value was calculated by a two-sided Wilcoxon test. The experiments were performed in two independent batches of differentiated neurons, with a total of 10 fields of view (FOVs) utilized for the quantification of each probe. The box plots in f, h and i display the medians, upper quartiles and lower quartiles; whiskers extending to 1.5× IQR; outliers are defined as ±1.5× IQR. For panels c-f, QTL P values were calculated by FastQTL linear regression. Source data

We next sought to understand how genetic regulation of APA might contribute to AD. First, we found that 336 (10.4%) AD-DAPAs also contained 3′aQTLs, but 3′aQTL and DAPA *P* values were not globally correlated (Extended Data Fig. 8a,b and Supplementary Table 7). Using colocalization and Mendelian randomization (MR), we found that 4 (of 75) genome-wide significant AD GWAS loci could have cell-type-specific APA regulatory mechanisms^68^ (Fig. 5a and Supplementary Table 16–18). Extending our analysis to subthreshold GWAS loci (5 × 10^−8^ < *P* < 1 × 10^−4^), we linked 10 of 337 loci to 3′aQTLs^69^ (Extended Data Fig. 8c,d and Supplementary Table 17). Altogether, we found 3′aQTL-driven explanations for 12 genes not previously linked to any QTL, including two mRNA processing genes, *SUPT4H1* and *SRSF10*^70,71^ (Fig. 5a). For example, *PLEKHA1*, which encodes a lipid-binding protein, showed 3′aQTL–pQTL–GWAS colocalization, consistent with its association with GWAS risk in a recent proteome study^66,72^ (Fig. 5b–d and Extended Data Fig. 8e–g).

Four GWAS-colocalized loci, including *SRSF10* and *SUPT4H1*, were also AD-DAPAs. Both genes showed different nuclear and cytoplasmic abundances of their proximal and distal 3′ UTR isoforms^56,73,74^ (Extended Data Fig. 8h). Using single-molecule inexpensive fluorescence in situ hybridization (smiFISH)^75^, we found significant differences in the localization of both genes’ proximal and distal isoforms in neurites versus somas of induced pluripotent stem cell (iPS cell)-induced neurons (Extended Data Fig. 8i, Supplementary Note and Methods). Together, these results suggest that some risk variants may contribute to AD by affecting 3′ UTR choice and thereby modulating the mRNA stability or localization of specific genes.

Finally, we identified a microglial 3′aQTL for *APOC2* within the *APOE* locus, whose lead SNP (rs7252480) was genome-wide significant in the AD GWAS, even after conditioning on 8 independent variants in the locus (*P* = 3.18 × 10^−11^)^76^ (Fig. 5a,e,f and Methods). We found no effect of rs7252480 on *APOC2* expression, indicating an eQTL-independent mechanism (Fig. 5g). Our results suggest that an *APOC2* 3′aQTL represents an *APOE-* and eQTL-independent contribution to AD risk.

### Cell-type-specific 3′aQTLs underlying brain traits

**Fig. 6:**
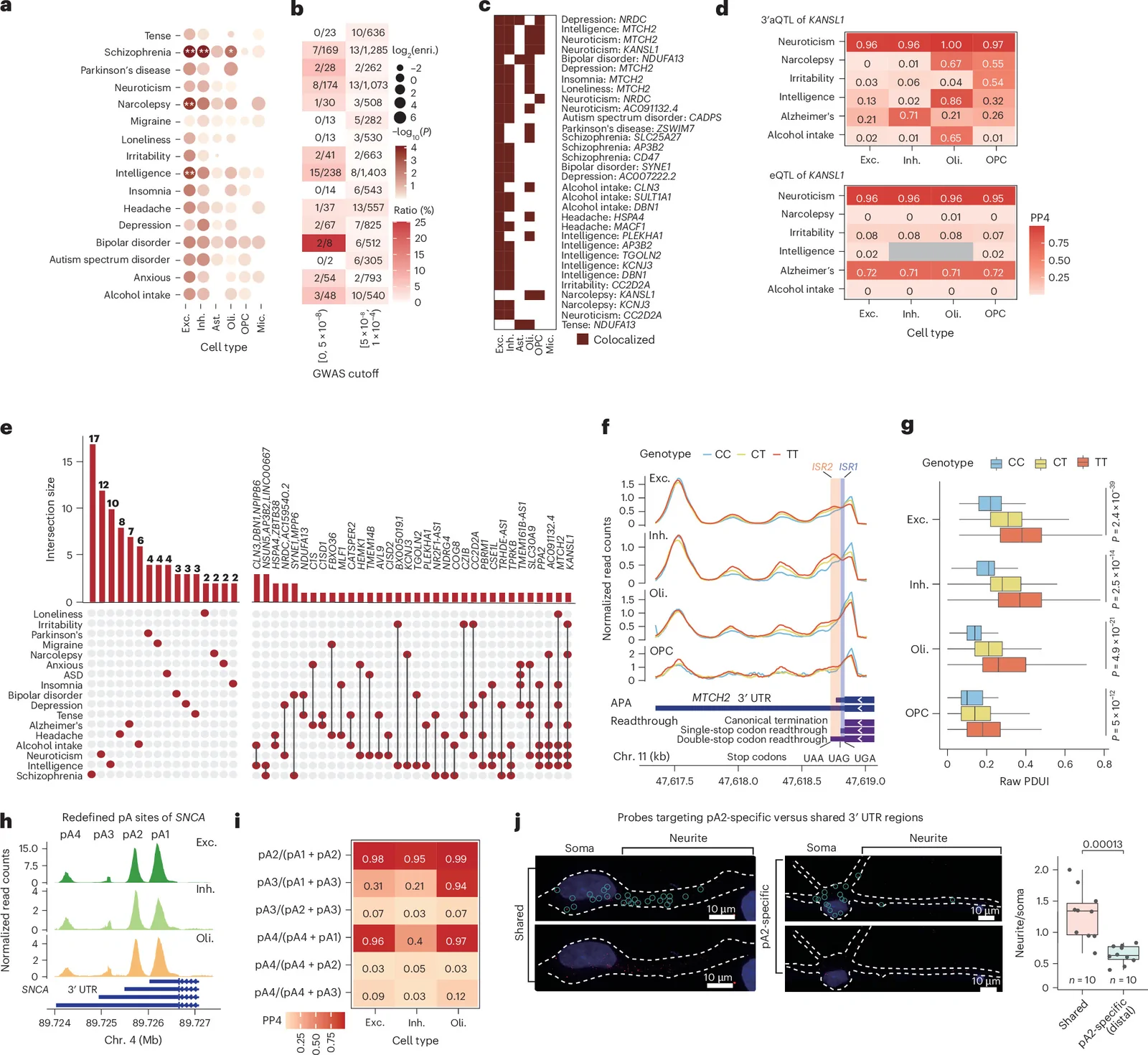
Cell-type-resolved 3′aQTLs interpret brain genetic disease loci. **a**, Heritability enrichment (LDSC) of 16 brain diseases or traits (rows) in 3′aQTLs in each cell type (columns). **b**, Proportions of GWAS loci of each trait colocalized with 3′aQTLs, for genome-wide significant (*P* < 5 × 10^−8^) or subthreshold (5 × 10^−8^ < *P* < 1 × 10^−4^) GWAS loci. *P* values denote the significance of enrichment as calculated by s-LDSC. **c**, GWAS loci (rows) colocalized with 3′aQTLs in at least two cell types (columns). **d**, A g-APA example showing both cell-type-sharing and cell-type-specific colocalization with different brain diseases. Colocalization posterior probabilities are shown for six traits at the *KANSL1* locus (rows) for cell-type-specific 3′aQTLs (columns, top) or eQTLs (bottom). Cell type–QTL combinations with no significant QTLs are shown in gray. **e**, UpSet plot showing numbers of 3′aQTL-colocalized disease-specific (left) or multidisease (right) GWAS loci, with multidisease gene names labeled. **f**, Normalized read count coverage of the *MTCH2* 3′ UTR for each rs34958982 genotype in four cell types (line chart). Bottom: long and short 3′ UTR isoforms (blue) and potential readthrough events (purple). Readthrough open reading frames are indicated by blue and red shaded rectangles. **g**, Per-genotype PDUI values of *MTCH2* for rs34958982 (boxplots) and 3′aQTL *P* values (right text) in four cell types (sample sizes: *n*_CC_ = 41, *n*_CT_ = 168 and *n*_TT_ = 136). *P* values were calculated by FastQTL linear regression. The box shows the 25th–75th percentiles, the line shows the median and whiskers show 1.5× IQR. **h**, Normalized coverage tracks across multiple cell types, showing four independent pA sites for the *SNCA* 3′ UTR. **i**, Colocalization posterior probabilities between PD GWAS at the *SNCA* locus and 3′aQTLs identified for different pairs of *SNCA* pA sites (rows) across different cell types (columns). **j**, smiFISH experiments showing soma versus neurite localization of *SNCA* isoforms in iPS cell-derived neurons. Left: representative cells with probes targeting pA2-specific and shared regions. Right: quantified neurite/soma ratios, with *P* values calculated by two-sided Wilcoxon test. The experiments were performed in two independent batches of differentiated neurons, with a total of 10 FOVs used for quantification of each probe. The box shows the 25th–75th percentiles, the line shows the median and whiskers show 1.5× IQR. ASD, autism spectrum disorder; enri., enrichment.

**Extended Data Fig. 9:**
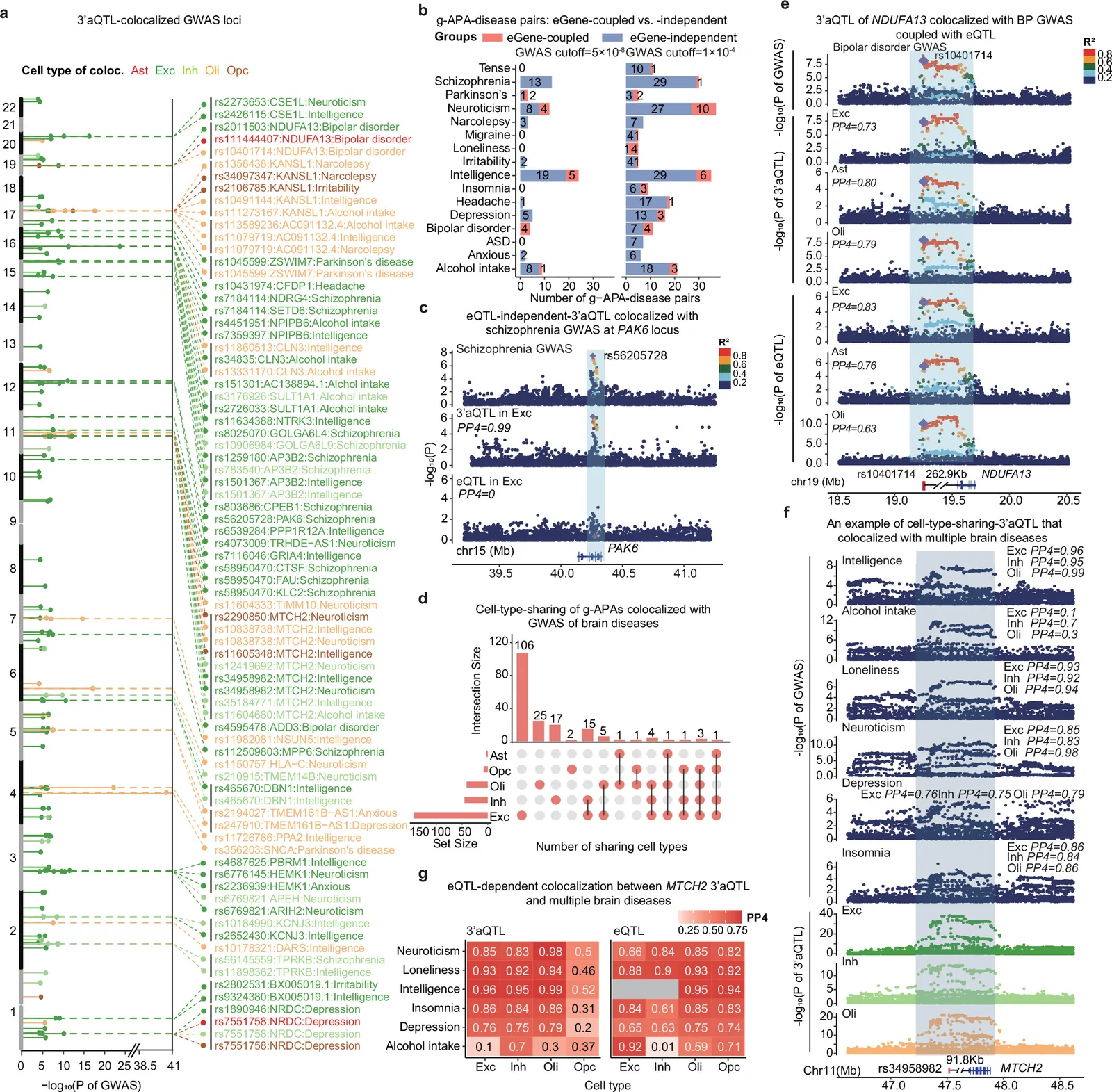
Colocalization between 3’aQTLs and GWAS loci of multiple brain diseases, related to Fig. 6. **a**, A Manhattan plot showing the GWAS P-values (x-axis) and the genomic position (y-axis) for the 3’aQTL-colocalized brain disease GWAS loci. P-values were reported by the original GWAS studies. **b**, Stack plot showing the numbers of 3’aQTL-GWAS colocalization loci that are eGene-coupled (orange) and eQTL-independent (blue) in 16 brain GWAS diseases. The GWAS loci with genome-wide (left) and subthreshold (right) significance were shown. **c**, Locus-compare visualization of *PAK6* locus as an example of eQTL-independent colocalization between 3’aQTLs and schizophrenia GWAS in excitatory neurons. QTL P values were calculated by FastQTL linear regression. **d**, UpSet plot showing the cell-type-sharing of the colocalization between 3’aQTLs and the GWAS loci of 17 brain diseases and traits (including AD). The barplot at the bottom left shows the numbers of colocalization in each cell type. **e**, Locus-compare plots showing the colocalization posterior probabilities between bipolar disorder GWAS and 3’aQTLs and eQTLs at the *NDUFA13* locus, which is an example of multi-colocalization across GWAS, 3’aQTL and eQTL. P values were calculated by FastQTL linear regression. **f**, Locus-compare plots showing the colocalization between the GWAS of multiple brain traits and the 3’aQTLs in multiple cell types at the *MTCH2* locus. P values were calculated by FastQTL linear regression. **g**, Heatmaps showing the colocalization posterior probabilities of the 3’aQTLs (left) and eQTLs (right) versus GWAS traits at the *MTCH2* locus (rows) in different cell types (columns).

Given the central role of 3′ UTRs in regulation of mRNA localization and stability in neurons^10,77^, we investigated whether our cell-type-resolved 3′aQTLs could explain heritability for 16 brain traits in the brain^78,79^ (Fig. 6a and Supplementary Table 16). Using stratified linkage disequilibrium (LD) score regression (s-LDSC)^80^, we found that schizophrenia heritability was enriched near 3′aQTLs from neurons and oligodendrocytes^81^ (Fig. 6a). At the level of specific risk loci, 45 genome-wide significant (*P* < 5 × 10^−8^; 4.69% of loci) and 109 subthreshold (5 × 10^−8^ < *P* < 1 × 10^−4^; 1.02% of loci) GWAS loci colocalized with 3′aQTLs across the 16 complex traits^25,26,82^ (Fig. 6b, Extended Data Fig. 9a,b and Supplementary Table 19). The vast majority of the 3′aQTL–GWAS-colocalized loci (79.5% of genome-wide significant loci and 82.5% of all loci) did not have colocalized eQTLs, consistent with previous reports (Extended Data Fig. 9b and Supplementary Table 20). For instance, schizophrenia-associated rs56205728, an eQTL-independent 3′aQTL associated with *PAK6*, encodes a protein kinase that has been implicated in brain development^83^ (Extended Data Fig. 9c).

Most GWAS-colocalized 3′aQTLs were identified in neurons (136 and 41), and 32 involved 3′aQTLs shared across cell types, with 24 shared between neuron types and 15 between neuronal and glial cells (Fig. 6c, Extended Data Fig. 9d and Supplementary Table 21). For bipolar disorder, we identified a multicell type 3′aQTL–eQTL (PP4: 0.63–0.83) for *NDUFA13*, a mitochondrial respiratory chain gene involved in cell death^84,85^ (Extended Data Fig. 9e). We observed trait-specific and cell-type-specific regulatory modes; for instance, the 3′aQTLs for *KANSL1* showed cell-type-specific colocalization in GWAS both with and without eQTLs, depending on the trait (Fig. 6d).

A large fraction of 3′aQTL-colocalized loci (*n* = 38, 31%) showed pleiotropic effects on multiple diseases (Fig. 6e). In contrast to the *KANSL1* locus, the *MTCH2* locus showed consistent 3′aQTL and eQTL colocalization with GWAS loci across both traits and cell types (Extended Data Fig. 9f,g). Notably, whereas 3′aQTL–eQTL-coupled colocalization might normally suggest APA-dependent gene expression alteration, *MTCH2* has the potential to have double stop codon readthrough^86,87^. The proximal *MTCH2* 3′ UTR is too short to contain the double readthrough’s final stop codon, suggesting that the 3′aQTL underlying the risk locus may directly alter the MTCH2 protein by modulating the relative amount of readthrough (Fig. 6f,g).

**Extended Data Fig. 10:**
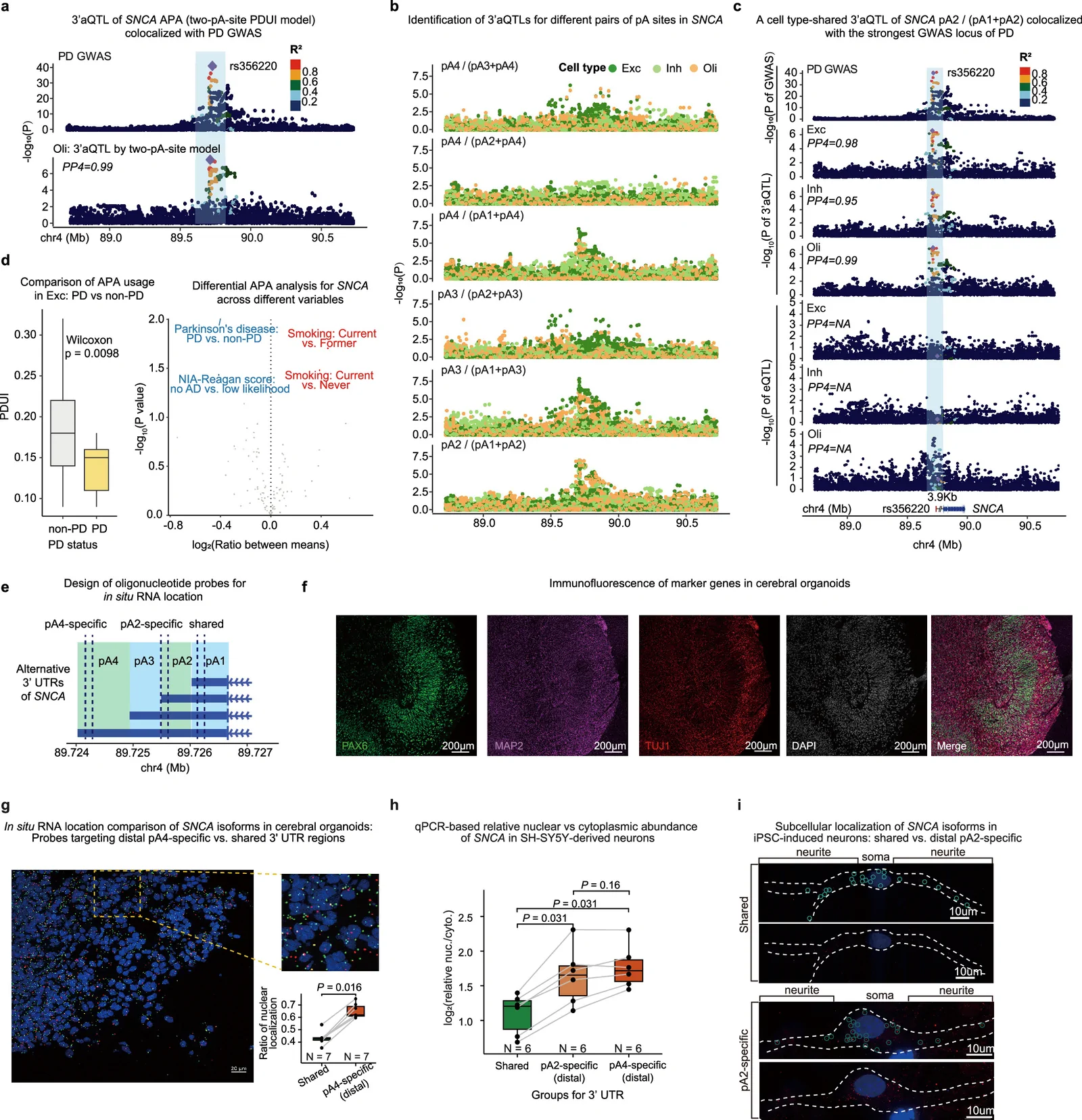
Contribution of SNCA APA in PD GWAS, related to Fig. 6. **a**, Colocalization between PD GWAS and two-pA-site-based 3’aQTLs at the *SNCA* locus in oligodendrocytes. **b**, Nominal P-values of 3’aQTLs for different pA site pairs of *SNCA* in excitatory and inhibitory neurons, and oligodendrocyte. **c**, Colocalization of PD GWAS with 3’aQTLs and eQTLs at the *SNCA* locus across excitatory neurons, inhibitory neurons, and oligodendrocytes. Colocalization posterior probabilities for each QTL with the PD GWAS are shown. In panels **a-c**, P values were calculated by FastQTL linear regression. **d**, *SNCA* PDUIs in excitatory neurons in individuals with and without Parkinson’s disease (non-AD individuals only: NIA-Reagan 3-4, N_non-PD_ = 133 and N_PD_ = 6) (left). Effect size (log_2_ ratio between means of PDUI distributions) versus -log_10_ p-value for biological variables for *SNCA* in excitatory neurons in non-AD individuals (NIA-Reagan 3-4) (right). P value was calculated using a two-sided Wilcoxon test. **e**, Design of oligonucleotide probes targeting pA4-specific-, pA2-specific, and shared *SNCA* 3’UTR regions. **f**, Immunofluorescence of day-40 cerebral organoids stained for PAX6, MAP2, TUJ1 and DAPI, where progenitor cells are defined by PAX6, and MAP2 and TUJ1 represent cortical plate neurons. Similar results were independently observed in six organoids. **g**, *In situ* hybridization of pA4-specific (red) and shared (green) *SNCA* 3’UTR regions in cerebral organoids (full image scale: 20μm; inset scale: 10μm). DAPI was stained for nuclei (blue). The boxplot shows the quantification of nuclear localization (right), with P value calculated by two-sided paired Wilcoxon test. Each point represents the average of three random fields of view from a single slice of one brain organoid (seven organoids in total, cultured in three batches). **h**, Relative nuclear versus cytoplasmic abundance of the isoform-shared, pA2-specific, and pA4-specific probes in SH-SY5Y-derived neurons. Significance was calculated by a two-sided paired Wilcoxon test. **i**, Representative FISH images showing soma versus neurite localization of *SNCA* isoforms in iPSC*-*derived neurons, with probes targeting pA2-specific and shared regions, respectively. The box plots of **d, g** and **h** display the medians, upper quartiles and lower quartiles; whiskers extending to 1.5× IQR; outliers are defined as ±1.5× IQR. Source data

We noticed that a neuronal and oligodendrocyte 3′aQTL was strongly colocalized (PP4 = 0.99) with the top locus for Parkinson’s disease (PD), near *SNCA* (GWAS *P* = 3.92 × 10^−41^)^26^ (Extended Data Figs. 9a and 10a). *SNCA* encodes α-synuclein, a key regulator of synaptic activity, aggregation of which is a pathogenic hallmark of PD^88–91^. To narrow down the part of the *SNCA* 3′ UTR driving PD risk, we separated it into four distinct pA sites and recalculated 3′aQTLs for the relative abundances of different pairs of these sites (Fig. 6h, Supplementary Note and Methods). Among these, 3′aQTLs involving the most proximal UTR (pA1) showed the strongest GWAS colocalization, suggesting that the switch between pA1 and distal pA sites mediates PD risk (Fig. 6i and Extended Data Fig. 10b,c). Notably, the lead variant (rs356220) showed little to no eQTL effect on *SNCA* expression, suggesting that the GWAS locus works through an expression-independent post-transcriptional mechanism mediated by APA regulation (Extended Data Fig. 10c). In addition, differential analysis revealed that *SNCA* had significantly more proximal 3′ UTR usage in excitatory neurons in individuals with PD (Extended Data Fig. 10d), whereas *SNCA* expression was not significantly altered in any major cell type (*P* = 0.18 for excitatory neurons). Notably, the decrease in PDUI in PD matched the effect direction of GWAS-colocalized 3′aQTL, as the PD risk allele also lowers the PDUI of *SNCA*^78^.

We next investigated the molecular consequences of proximal *SNCA* 3′ UTR usage. FISH imaging in brain organoids revealed significantly lower cytoplasmic (versus nuclear) abundance of the longest *SNCA* isoform (pA4) relative to the shortest isoform (pA1) (Extended Data Fig. 10e–g and Methods). Similarly, distal *SNCA* isoforms (pA2 and pA4) were significantly depleted in the cytoplasmic (versus nuclear) fraction of SH-SY5Y-derived neurons according to quantitative PCR analysis (Extended Data Fig. 10h and Supplementary Note). Using smiFISH^75^ in iPS cell-derived neurons, we found that the pA1 isoform preferentially localized to neurites, whereas the pA2 isoform was more abundant in soma (Fig. 6j and Extended Data Fig. 10i). Together, these results suggest that the top PD risk locus acts on *SNCA* through a 3′aQTL that promotes its proximal 3′ UTR usage, thereby altering neuronal localization of *SNCA* mRNA.

## Discussion

Here we report a cell-type-resolved APA landscape across hundreds of human brains, elucidating DAPA usage across cell types, AD status and genetic variation. We integrated 3′aQTLs with eQTLs and pQTLs to interpret GWAS loci mediated through APA, providing insights into a crucial regulatory layer for understanding complex human disease.

Our results shed light on cell type differences in 3′ UTRs across neuronal and glial cells at both global and locus-specific levels and propose underlying *cis*-regulatory elements and *trans*-acting regulators for APA. We found that the global differences in 3′ UTR lengths were mainly driven by different regulatory environments rather than cell-type-specific gene expression. We identified and experimentally validated multiple potential APA regulators, including *PCBP2* and *U2AF2*, knockdown of which induced reproducible APA changes that were consistent with their predicted targets in single-cell data. These genes are primarily known as spliceosome components, but our work suggests that they may also participate in APA regulation.

We also identified a pronounced alteration of APA in the late stage of AD, with more than 3,000 AD-DAPA events across all cell types. Neurons accounted for the majority of altered 3′ UTRs in AD, and this was only partially explained by increased statistical power. Neuronal genes with increased 3′ UTR lengths during AD progression were highly enriched in microglial cell activation, microtubule-related transport and axodendritic transport pathways, dysregulation of which has been linked to the etiology and progression of AD^1,49,92^. Notably, the vast majority of genes with AD-DAPAs showed no expression change in AD; these included key AD risk genes such as *MAPT*, *ABCA7* and *MEF2C*. Overall, our results suggest that APA is an important and complementary regulatory layer that should be fully mapped to provide a complete understanding of the biological mechanisms underlying AD and other brain diseases.

We identified 3′aQTLs for 4,288 genes across 6 brain major cell types. We demonstrated that 3′aQTLs colocalize more frequently with pQTLs than with eQTLs. Thus, 3′aQTLs may be a valuable target for future investigation, as they are orthogonal to eQTLs and overlap with a substantial fraction of pQTLs, which are currently much harder to profile at scale. Of note, the cell type eQTLs were measured based on snRNA-seq data, which may not entirely represent the expression dynamics in the whole cell. Expression differences resulting from 3′ UTR changes that affect mRNA stability may not be detectable in the nucleus; this may have been partly responsible for the relatively low colocalization between eQTLs and 3′aQTLs^93^.

Our cell-type-resolved 3′aQTLs shed light on GWAS variants for AD and other brain traits. About 4.7% of loci across 17 brain traits and diseases were colocalized with a 3′aQTL, and the majority (82.5%) of 3′aQTL–GWAS-colocalized loci did not also colocalize with an eQTL. Notably, although AD heritability is mainly enriched in microglial regulatory elements, most AD-associated 3′aQTLs were in neurons, suggesting that AD heritability may be partly mediated through neuronal APAs. Furthermore, conditional analyses of the *APOE* locus uncovered *APOC2* APA as an independent AD GWAS hit, consistent with recent reports of multiple causal signals within this locus^76^. Finally, our results suggest that the top PD GWAS risk locus alters *SNCA* 3′ UTR choice across multiple pA sites, which may subsequently affect the cellular localization or compartment-specific stability of *SNCA* mRNA in neurons. Further work is warranted to fully link the PD risk locus to *SNCA* mRNA localization and to understand its downstream functional effects. This locus provides an example of how disease risk variants can act through APA without changing mRNA or protein levels and could serve as a model for study of other risk loci across complex diseases.

Our study had several limitations. First, although the identified pA sites were strongly enriched for canonical polyadenylation signals, the enrichment was slightly lower than for database-curated sites^1,32^, probably owing to both technical noise in sparse snRNA-seq data and biological heterogeneity across cells. Second, although we predicted and validated various APA regulator candidates, further experiments could help to distinguish direct from secondary effects. Third, statistical power for identifying AD-DAPAs and 3′aQTLs was limited for nonneuronal cell types. These limitations could be mitigated by increased coverage, biased profiling to recover rare cell types and direct 3′ UTR profiling.

Overall, our study provides a resource for understanding the landscape and genetic regulation of APA in the brain and indicates how future studies of development and disease could tap single-cell transcriptomic datasets to interrogate a critical regulatory layer with unique molecular consequences. As APA is largely orthogonal to gene expression, we propose that this accessible and key post-transcriptional layer should be mapped as a complementary modality in future single-cell studies to provide a comprehensive picture of gene regulation in health and disease.

## Methods

### snRNA-seq data collection

The results published here are in whole or in part based on data obtained from the AD Knowledge Portal (https://adknowledgeportal.org/). Study data were generated from postmortem brain tissue provided by the ROSMAP cohort at Rush Alzheimer’s Disease Center, Rush University Medical Center, Chicago. We used the snRNA-seq data of the prefrontal cortex from a subset (*n* = 379; deposited in the ‘Sequencing Round 2’ folder accessible via https://www.synapse.org/Synapse:syn52362197) of ROSMAP participants whose postmortem prefrontal cortexes we had previously profiled^18^. We selected only the 379 samples that were profiled using Chromium Single Cell 30 Reagent Kits v.3, and not those profiled with the v.2 chemistry, to minimize technical artifacts arising from use of different protocols. We used the same major cell-type-level cell annotations as the original study. The individuals were grouped into non-AD (*n* = 197), early AD (*n* = 121) and late AD (*n* = 61) categories according to *k*-means clustering of 7 pathological measurements, as in ref. ^94^. Clinical and pathological procedures have been previously reported^95–99^. ROSMAP resources can be requested via https://www.radc.rush.edu and www.synapse.org.

### Identification of pA sites and transcriptome-wide 3′ UTR length comparison

We carried out de novo pA site identification at single-cell level using SCAPE^100^. For consistency with the snRNA-seq reads alignment, we used the GENCODE (v.32) gene annotation of the GRCh38 genome to identify pA sites. We calculated the enrichments of the AWTAAA motif in flanking sequences of these pA sites. Specifically, we used DREME (v.5.5.4) to search for enriched motifs within the 50-bp sequences upstream of the pA sites; the identified A[A/T]TAAA motif was subsequently used as an input to FIMO to locate the position of the motif relative to the pA sites^101^. We estimated the global 3′ UTR length across all the genes per cell by computing the 3′ UTR length for each pA site by calculating the distance between the pA site and the annotated stop codon, with introns excluded. Details of the calculation and analysis methods are provided in Supplementary Note.

### Two-pA-site-based quantification of APA

To facilitate 3′aQTL identification and DAPA analyses, we used a 2-pA-site model, DaPars2^25^. We first generated pseudobulk alignment files for each cell type in each sample, based on the major cell type cell annotation^18^. For each gene in each pseudobulk sample, we identified proximal and distal pA sites using DaPars2 and then calculated a PDUI (defined as the ratio between the number of distal reads and the sum of proximal and distal reads). A larger PDUI value represents a larger fraction of the long version of 3′ UTR.

### APA quality control and matrix imputation

To ensure high quality of the APA matrix, we excluded genes that were not detected in more than 50% of individuals for each cell type. The resulting APA matrix for each cell type was subsequently sent for imputation using the complete function of R package mice (v.3.16.0)^102^; this function performs multiple imputations for multivariate missing data, with each incomplete variable imputed by a separate model based on fully conditional specification^103,104^.

### APA *trans* regulator detection

We prioritized potential *trans* regulators for global 3′ UTR length based on the Spearman correlation of the expression of the regulator and the global 3′ UTR length across samples. We only considered RBPs or TFs as potential regulators, as annotated in the POSTAR3 database^105^. We calculated Spearman correlation coefficients and *P* values, adjusted *P* values using the Bonferroni method, and kept RBPs and TFs with adjusted *P* < 0.01, considering the effects of age at death, sex and postmortem interval (PMI).

### RNA-seq of SH-SY5Y-derived neurons with gene knockdown

To validate candidate APA regulators, we performed gene knockdown in SH-SY5Y-derived neuronal cells (experimental details are provided in Supplementary Note). Oligonucleotides for knockdown are listed in Supplementary Table 22. Total RNA of the SH-SY5Y-derived neuronal cells with gene knockdown was collected using an AllPure DNA/RNA/Protein CW0591S kit (CWBIO) according to the manufacturer’s instructions. The corresponding cDNA library was sequenced using an Illumina NovaSeq 6000 (Repugene Technology).

APA usage was quantified using DaPars2 with Coverage_threshold = 80 to generate PDUI values. To confirm reproducibility between short hairpin RNAs and technical replicates, we calculated Pearson correlation coefficients across samples based on global mRNA expression and APA profiles and performed clustering using these coefficients, with expression and APAs normalized by the corresponding nontarget control samples. We calculated the difference and fold change in PDUI between knockdown and nontarget samples for each gene. Genes with absolute value of log_2_(fold change) greater than 0.25 were defined as potential targets upon gene knockdown.

### Cell-type-specific APA analysis

To mitigate APA detection bias caused by cell numbers and sequence depth, we kept only genes with APA events in at least four cell types for cell-type-specific APA detection. We calculated the difference in PDUI between the tested cell type and the remaining five cell types using Wilcoxon tests and used the false discovery rate (FDR) to account for multiple APAs tested. Genes with FDR < 0.05 were defined as potential cell-type-specific APA genes. Given the high similarity between excitatory and inhibitory neurons, we further defined genes that were lengthened or shortened in both excitatory and inhibitory neurons as neuron-specific APA genes. To understand the biological pathways of these cell-type-specific APA genes, we performed gene ontology (GO) enrichment analysis for the lengthened or shortened APA genes for each cell type using clusterProfiler^106^. To ensure that we identified the pathways underlying the AD-DAPA genes rather than the general pathways of the corresponding cell types, we used genes with APAs in the relevant cell type as the background set and kept GO terms with *P* < 0.01. To avoid showing redundant GO terms in the figures, we iteratively removed GO terms whose underlying genes overlapped by more than 50% with those of a more significant GO term. Last, the differential 3′ UTRs generated by cell-type-specific lengthened or shortened APAs were used for motif enrichment analysis using the Simple Enrichment Analysis function of the MEME toolkit^101^; the RBPs with *P* < 0.05 and enrichment ratio > 1 are shown in Extended Data Fig. 2g.

### Identification of AD-DAPA genes

We used transcript-level PDUI values to identify AD-DAPAs using both regression-based and pairwise nonparametric approaches. We regressed out sex, age at death and PMI from PDUI values and used the residuals to identify AD-DAPAs for both approaches. Nonvariable genes (PDUI = 0 or 1) in more than 80% of samples were excluded. For the regression approach, we numerically encoded the three AD progression groups as 0, 1 and 2, for non-AD (*n* = 197), early AD (*n* = 121) and late AD (*n* = 61), respectively; then, we calculated Spearman’s rank correlation coefficients and *P* values for the numeric AD progression for each transcript in 6 major cell types and defined regression-based AD-DAPAs as genes with absolute correlation > 0.15 and FDR < 0.1. For the pairwise or stage-specific approach, we used Wilcoxon tests to compare gene-level PDUIs for non-AD versus early AD, early AD versus late AD, and non-AD versus late AD; then, we defined pairwise AD-DAPAs as genes with absolute log_2_(fold change) > 0.15 and FDR < 0.1. Finally, we defined the final set of significant AD-DAPAs as genes that were DAPAs in both the regression and pairwise approaches. Results obtained with and without accounting for covariates are compared in Extended Data Fig. 3c.

We performed GO functional enrichment analysis on DAPA genes using clusterProfiler^106^. We used genes with APA in the relevant cell type as the background set and kept GO terms with *P* < 0.01. To avoid showing redundant GO terms in the figures, we iteratively removed GO terms whose underlying genes overlapped by more than 50% with those of a more significant GO term. The full list of DAPA-enriched GO terms is provided in Supplementary Table 9.

### Identification and removal of confounding factors

To mitigate the confounding effects of nongenetic factors on 3′aQTL identification, we included sex, age at death and PMI as covariates into the 3′aQTL identification model. To correct for the effects of population stratification, we performed principal component analysis on the genotype matrix using gcta64^107,108^. The top 5 genotype principal components were considered as covariates for 3′aQTL identification. In addition, we detected confounding factors using PEER (R package 1.0) for the APA matrix in each cell type^109^. Finally, based on the number of g-APAs identified, we selected PEER factors 2–11 for excitatory neurons, 1–8 for inhibitory neurons, 1–6 for astrocytes, 1–8 for oligodendrocytes, 1–7 for OPCs and 1–5 for microglia.

### 3′aQTL identification

For SNP quality control, we first restricted the analysis to individuals with both genotype and single-cell data available. Among these overlapping samples, SNPs with minor allele frequency < 0.01 were removed using PLINK2, and the remaining SNPs were used for 3′aQTL identification^110^. For PDUI matrix processing, we first removed genes with no variation across all the individuals in each cell type. We then used the Voom function to normalize the PDUI matrix in each cell type^111^. The normalized PDUI matrices were used for 3′aQTL identification. We used FastQTL (v.2.184) for 3′aQTL detection with the covariates described above^111,112^. We tested the association between the normalized PDUI and all SNPs within 1 Mb distance for each APA. We carried out two rounds of multitesting correction to account for the multiple SNPs tested for each APA gene and the multiple APA genes tested. To define g-APA genes, we used 1,000 permutation tests to identify the lead SNP for each APA and to calculate the adjusted (empirical) *P* value. Subsequently, the empirical *P* values of the lead SNPs across all the APAs were subjected to a second round of correction using *q*-values. APAs with *q* < 0.05 at their lead SNPs were defined as significant g-APAs.

### Genomic enrichments of 3′aQTLs

To understand the genomic distribution preference of 3′aQTLs, we first used HOMER to annotate the genomic locations of the lead 3′aQTLs^113^, assigning each 3′aQTL to one of the following regions: 3′ UTR, transcriptional terminal site, transcription start site, exon, intron, intergenic, promoter and 5′ UTR. We then carried out a hypergeometric test to evaluate the relative enrichments of 3′aQTLs across these regions. This analysis was performed by combining the 3′aQTLs across all the cell types to ensure robustness. The genomic enrichments with nominal *P* < 0.01 and fold change > 2 were considered to be significant.

### Comparison between 3′aQTLs from snRNA-seq and bulk RNA-seq

To evaluate the consistency of 3′aQTLs identified at the cell type level versus the bulk level, we compared the overlap of g-APAs and the effect size correlations of the 3′aQTLs identified by this study and by bulk brain studies^25,26^. As different gene annotations were used in different studies, we used R package org.Hs.eg.db to convert gene IDs and enable direct comparison. The genes that were used for 3′aQTL identification by both studies were used for the g-APA overlap and 3′aQTL effect size comparison. We first calculated the number of intersections of g-APAs identified in this study and in the other studies, including GTEx brain cortex, ROSMAP and PsychENCODE. We then used the GTEx brain cortex dataset, which reported the 3′aQTL effect size^25^, to evaluate the correlation and directionality consistency of the lead 3′aQTL effect size for the shared g-APAs between two datasets. Directionality consistency was calculated as the number of 3′aQTLs showing the same sign of effect size (either positive or negative) divided by the number of shared g-APAs.

### 3′aQTL comparison across cell types

We carried out a direct overlap of g-APAs to evaluate the sharing of 3′aQTLs across cell types. In addition, we used a previously reported method based on directionality consistency of 3′aQTLs^60,61^. For the lead 3′aQTLs that were significant in at least one cell type (discovery cell type), we determined whether they showed the same directionality effect of 3′aQTL in each of the remaining cell types (replication cell types). Directionality consistency was then quantified as the percentage of 3′aQTLs that showed consistent effect sizes between discovery and replication cell types. To estimate the consistency of 3′aQTLs under different significance levels in replication cell types, we grouped the 3′aQTLs based on their nominal *P* values in the replication tissues, including the following groups: [0, 1 × 10^−8^), [1 × 10^−8^, 1 × 10^−6^), [1 × 10^−6^, 1 × 10^−4^), [1 × 10^−4^, 0.01), [0.01, 0.1), [0.1, 0.5) and [0.5, 1). Pairwise sharing of 3′aQTLs between cell types (Fig. 3f) was quantified as:

$$\mathop{\sum }\limits_{i=1}^{{n_{\mathrm{bin}}}}{\mathrm{proportion}}_i\times [{\mathrm{consistency}}_i-(1-{\mathrm{consistency}}_i)],$$

where ${\mathrm{proportion}}_i$ represents the proportion of 3′aQTLs in *P* value bin *i*, and ${\mathrm{consistency}}_i$ denotes the 3′aQTL directionality consistency in *P* value bin *i*. The quantified pairwise between-cell-type consistency was further used to carry out hierarchical clustering across cell types.

### 3′aQTL-dependent APA regulator identification

To predict potential 3′aQTL-dependent APA regulators, we performed enrichment analysis of 3′aQTLs in the binding sites of RBPs or TFs. We first downloaded the eCLIP–seq binding sites of RBPs and the ChIP–seq binding sites of TFs profiled in two cell lines (K562 and HepG2) from ENCODE^62,114^ and merged the peaks from the two cell lines as the final binding sites. We examined the expression of the genes encoding the tested RBPs and TFs in the 3′aQTL discovery cell type; only the expressed RBPs and TFs were used for the enrichment analysis. We next used the significant 3′aQTLs in each cell type to assess enrichment against the eCLIP–seq and ChIP–seq binding sites using Torus^115^. RBPs and TFs with enrichment log(OR) > 0.5 and FDR < 0.05 were considered to be potential 3′aQTL regulators. We then evaluated the regulators using two additional metrics to increase confidence in their identification. First, we determined Spearman correlations between the gene expression of the regulators and the PDUIs of their targeted APAs; regulators with Bonferroni-adjusted *P* values of correlation less than 0.05 were considered to be significant. Second, we curated known APA regulators^1^ and constructed a protein–protein interaction network of known and predicted APA regulators using the STRING database^116^.

### Integrative analysis between 3′aQTLs, eQTLs and pQTLs

We first performed cell-type-resolved eQTL identification using the same dataset as for 3′aQTL identification. We took a similar pipeline to that used for 3′aQTL identification, including gene expression matrix normalization using Voom, confounding factor identification using PEER, and eQTL identification with covariates removed using FastQTL. We investigated the relationships of the intersecting eGenes and g-APA genes using two approaches: first, we calculated the lead variant distance between 3′aQTLs and eQTLs; second, we performed colocalization analysis between eQTLs and 3′aQTLs using coloc^117^. Pairs with PP4 > 0.1 and PP4/(PP4 + PP3) > 0.75 were considered to be significant.

To systematically explore the relationship across 3′aQTLs, eQTLs and pQTLs, we downloaded a pQTL dataset profiled in brain tissue^66^ and conducted colocalization analyses. The proportion of colocalization between each pair of QTL types was calculated as the number of colocalized loci divided by the number of loci tested. We used R package locuscomparer to visualize the colocalized examples, plink2 to calculate LD correlations between SNPs and used R package LD heatmap to visualize LD heatmaps^118,119^. We conducted Fisher’s exact tests to examine whether the overlap between pQTLs and 3′aQTLs was significantly higher than that of eQTLs and 3′aQTLs. Specifically, Fisher’s exact test was performed on a 2-by-2 matrix (*a*, *b*, *c*, *d*), where *a* is the number of 3′aQTL–pQTLs tested that did colocalize, *b* is the number of 3′aQTL–pQTLs tested that did not colocalize, *c* is the number of 3′aQTL–eQTLs tested that did colocalize and *d* is the number of 3′aQTL–eQTLs tested that did not colocalize.

### Integrative analysis between 3′aQTLs and AD GWAS

We used colocalization and MR to identify g-APA genes potentially involved in AD GWAS, focusing on both genome-wide significant (*P* < 5 × 10^−8^) and subthreshold (5 × 10^−8^ < *P* < 1 × 10^−4^) GWAS loci. We used the coloc package (v.5.2.3) for the colocalization analysis, and 3′aQTL–GWAS pairs with PP4 > 0.5 were defined as significant colocalized loci. The MR analysis was carried out using the TwoSampleMR package (v.0.5.6). In the MR analysis, 3′aQTLs were used as instrumental variables (IVs) in exposure. We performed LD clumping to ensure the independence of IVs using plink (*r*^2^ < 0.01, window size: 100 kb). We used the inverse variance weighted regression method for pairs with multiple independent IVs and the Wald ratio method for g-APAs with a single IV. *P* values of the MR outputs were corrected using the Benjamini–Hochberg method, and loci with corrected *P* values less than 0.05 were defined as significant.

### Conditional analysis of *APOC2* locus in AD GWAS

Given that *APOC2* was located near *APOE*, which is the strongest AD GWAS locus, we performed conditional analysis to evaluate the independence of the heritability of *APOC2* on AD. As the *APOE* locus was excluded from the most recent AD GWAS study, we used the dataset from an earlier study to perform a conditional analysis for the *APOC*2 locus^68,76^. We used six variants (rs41289512, rs1081105, rs6859, rs28399637, rs7412 and rs10407439) previously reported to be independently associated with AD^76^, as well as two independent variants (rs76856627 and rs117612135) near the *APOC*2 locus, to perform AD GWAS conditional analysis. Another reported AD-associated variant, rs138607350, was not included for the analysis because of its low minor allele frequency (<0.01) in our cohort. GCTA was utilized for the conditional analysis, with 1000 Genomes as the reference panel^108^. To mitigate the effects of other independent loci and enable us to more accurately assess the colocalization effect, we reperformed colocalization analysis between 3′aQTLs and AD GWAS for the *APOC2* locus after conditional analysis.

### Cell-type-resolved heritability enrichment and colocalization analysis of 3′aQTLs in GWAS

We assessed the heritability enrichments of the 3′aQTLs identified in each cell type against 17 brain GWAS traits (Supplementary Table 16). The heritability enrichment analysis was performed using s-LDSC (v.1.0.1), with baselineLD v.1.1 (provided by s-LDSC) used as the baseline model^80^. To further detect brain disease GWAS loci mediated by 3′aQTL, we performed colocalization analysis between 3′aQTLs in each cell type and these brain GWAS traits using the same procedure as for the integrative analysis with AD GWAS. We used GWAS variants at both genome-wide (*P* < 5 × 10^−8^) and subthreshold (5×10^−8^ < *P* < 1 × 10^−4^) significance. To quantify the proportion of independent loci interpretable by 3′aQTLs for each GWAS, we conducted LD clumping for the GWAS loci for each trait, with clump-r2 and clump-kb set to 0.005 and 500, respectively. For each GWAS trait, we calculated the proportion of independent GWAS loci interpretable by 3′aQTLs by dividing the number of GWAS loci colocalized by at least one g-APA locus by the total number of independent loci.

### Analysis of the *SNCA* locus in PD GWAS

As we found multiple pA sites in the *SNCA* 3′ UTR based on visualization of raw sequencing coverage, we carried out high-resolution pA site identification. We first split the *SNCA* 3′ UTR into 10-bp bins, calculated the average sequence depth of each bin in each cell type, and used the rollapply function of R package zoo (v.1.8-12) to scale and smooth the average sequence depth. We then calculated the difference between each adjacent bin pair; a bin showing a negligible difference (<0.01) compared with its adjacent bin was defined as the start or the end of a pA site peak (Fig. 6h). We performed pairwise pA site usage index analysis, and then reperformed 3′aQTL identification using the same procedure for each pA site pair in *SNCA*. The resulting 3′aQTLs for each pA site pair were further used to perform colocalization analysis with PD GWAS at the *SNCA* locus.

### smiFISH for subcellular localization investigation

To study the subcellular localization of different APA isoforms, we performed smiFISH in iPS cell-derived neurons based on a protocol described by Tsanov et al.^75^. This method allows visualization of individual RNA molecules through a two-part probe design, in which gene-specific primary probes are hybridized to a short universal secondary probe conjugated with a fluorophore. For experimental details, see Supplementary Note.

For each probe, a total of 10 fields of view (FOVs) were selected from two independent batches of differentiated neurons as replicates for the quantitative analysis. Within each FOV, we calculated the fold change between RNA molecules detected in neurites versus soma for each cell; cells that showed no detectable signal in either neurites or soma were excluded. Then, we aggregated the fold changes between neurites versus soma for cells within each FOV and used Wilcoxon tests to evaluate the significance of the differences of these fold changes between 3′ UTR-shared versus distal-pA-specific groups for each gene.

### Reporting summary

Further information on research design is available in the Nature Portfolio Reporting Summary linked to this article.

## Online content

Any methods, additional references, Nature Portfolio reporting summaries, source data, extended data, supplementary information, acknowledgements, peer review information; details of author contributions and competing interests; and statements of data and code availability are available at https://doi.org/10.1038/s41588-026-02758-w.

## Supplementary information

Supplementary Information Supplementary Note and References.

Reporting Summary

Supplementary Tables 1–25 Supplementary Table 1: individual-level metadata table. Supplementary Table 2: cell-type-resolved APA events identified in this study. Supplementary Table 3: APA regulator identification based on regression analysis. Supplementary Table 4: targets of predicted APA regulators in knockdown experiments. Supplementary Table 5: cell-type-specific APAs in each cell type. Supplementary Table 6: RBPs with binding site enrichments in cell-type-specific APA regions. Supplementary Table 7: AD-differential APAs in each cell type. Supplementary Table 8: AD-differential gene expression in each cell type based on regression analysis. Supplementary Table 9: significant GO terms enriched by AD-differential APA genes. Supplementary Table 10: significant GO terms enriched by AD-DEGs. Supplementary Table 11: g-APAs and the corresponding lead SNPs in each cell type. Supplementary Table 12: cell type sharing of g-APA genes across cell types. Supplementary Table 13: predicted 3′aQTL-dependent APA regulators. Supplementary Table 14: eGenes and the corresponding lead SNPs in each cell type. Supplementary Table 15: colocalization analysis across 3′aQTL, eQTL and pQTL. Supplementary Table 16: information of disease GWAS used in this study. Supplementary Table 17: AD GWAS loci potentially mediated by 3′aQTLs. Supplementary Table 18: colocalization analysis between eQTL/pQTL and AD GWAS. Supplementary Table 19: colocalization analysis between 3′aQTL and brain disease GWAS. Supplementary Table 20: colocalization analysis across 3′aQTL, eQTL and brain disease GWAS. Supplementary Table 21: cell type sharing of 3′aQTL-colocalized GWAS loci. Supplementary Table 22: primers used in short hairpin RNA knockdown experiments for APA regulators. Supplementary Table 23: primers of CRISPR-based prime editing in 3′aQTL validation. Supplementary Table 24: quantitative PCR primers for nuclear cytoplasmic difference experiments. Supplementary Table 25: oligonucleotide sequences for FISH experiments.

## Source data

Source Data Fig. 1 Statistical source data.

Source Data Extended Data Fig. 1 Unprocessed western blots.

Source Data Extended Data Fig. 4 Unprocessed western blots and statistical source data.

Source Data Extended Data Fig. 8 Statistical source data.

Source Data Extended Data Fig. 10 Statistical source data.

## Data Availability

The snRNA-seq data are available via Synapse under accession syn52293417. Summary statistics for the 3′aQTLs and eQTLs generated in this study are available via GitHub at https://cboix.github.io/snAPA and Zenodo at https://doi.org/10.5281/zenodo.20740673 (ref. ^120^). The RNA-seq data for the knockdown experiments for regulator validation are available via the National Genomics Data Center under accession HRA014304. Source data are provided with this paper.

## Code availability

Analysis code is available via GitHub at https://github.com/xiongxslab/NanJ_Carles_et_al_3aQTL_project/ and Zenodo at https://doi.org/10.5281/zenodo.20740673 (ref. ^120^).
