# Supplementary material for: Single-cell profiling and genetic regulation of alternative polyadenylation in the human brain
Source: Nat Genet. 2026 Sep 30;58(11):2819–31. doi: 10.1038/s41588-026-02758-w (PMC13754597; doi:10.1038/s41588-026-02758-w)
Supplement: Supplementary file 1 [file 41588_2026_2758_MOESM1_ESM.pdf]

nature genetics

Supplementary information

https://doi.org/10.1038/s41588-026-02758-w

# Single-cell profiling and genetic regulation of alternative polyadenylation in the human brain

In the format provided by the authors and unedited

## Supplementary Note

### Estimation of the global 3’UTR length and relevant comparison

For the comparison of global 3'UTR length across all the genes, we first computed the 3'UTR length for each pA site by calculating the distance between the pA site and the annotated stop codon with introns excluded. For each gene in each cell, we calculated a mean 3'UTR length across all the pA sites of that gene, weighted by the supporting counts of each pA site. We then calculated a “scaled 3'UTR length” for each gene in each cell, by subtracting the 3'UTR length in that cell from the mean of 3′ UTR lengths across all the cells. Subsequently, for each cell, the global 3'UTR length can be calculated as the mean of the scaled 3'UTR length across all the genes in that cell. We defined the genes expressed in at least four of the seven major brain cell types as the broadly expressed genes. We compared the 3'UTR length across cell types by aggregating 3'UTR length across all the cells per cell type, both for all genes and broadly expressed genes.

### Evaluate the impact of technical factors on 3’UTR length by downsampling

To evaluate the effects of cell number on 3'UTR length, we downsampled the data to 20, 30, or 40 cells per cell type and individual and recalculated 3'UTR lengths. To assess the impact of cell-level technical covariates on 3'UTR length, we binned cells into quantiles according to five covariates and calculated 3'UTR lengths in each quantile. These covariates are the number of detected genes (n_genes), detected 3'UTRs (n_3'UTRs), sequencing read count (n_counts), sequencing read count per detected gene (n_counts per gene) and sequencing read count per detected 3'UTR (n_counts per 3'UTR) in each cell.

### Evaluate the impact of technical factors on AD-differential APA genes by downsampling

To evaluate the impact of cell number and sequencing depth on AD-DAPA identification, we performed downsampling analysis on cell number and read count. Given the limited cells and sequencing depth of microglia, we excluded this cell type in downsampling analysis to ensure the range of downsampling across other cell types. Firstly, we used samtools[1] (Version: 1.10) to generate downsampling bam files. Then, based on downsampling bam files, we identified proximal and distal pA sites using DaPars2, calculated a PDUI, and detected AD-differential APA genes using the same parameters as the above analysis. For the downsampling on sequencing depth, we randomly selected 40, 80 and 100 cells per cell type per individual for 10 times, and the 238 individuals with more than 100 cells across all the cell types were used for this analysis. For the downsampling on read count, we performed downsampling analysis for both five cell types (excluding microglia and vascular cells) and two neuronal cell types. For each of the five cell types, we randomly extracted 100k, 500k and 1M reads across 198 individuals for 10 times. For neuronal cell types, we randomly extracted 3M and 5M reads of 190 individuals for 10 times.

### Experimental validation of 3’aQTLs

We selected 3’aQTLs for experimental validation based on: (i) the significance level of each locus, (ii) LD between the tested SNP and the lead variant, and (iii) proximity to the “A(A/T)TAAA” polyadenylation signal. Finally, we selected two g-APA loci for validation using CRISPR prime editing in HEK293T cells. Specifically, prime editing was performed using the PE3 system[2]. PegRNA and nickRNA sequences were designed using the pegFinder tool (http://pegfinder.sidichenlab.org/). The pegRNA spacer, scaffold, and template sequences were synthesized as single-stranded oligonucleotides, annealed, and ligated into pegRNA and epegRNA acceptor plasmids (pU6-tevopreq1-GG-acceptor) via Golden Gate assembly.

Briefly, the pU6-tevopreq1-GG-acceptor plasmid was digested with BsaI-HFv2, NcoI-HF, and PvuII-HF, and the resulting 2.2 kb restriction fragment was purified using the QIAquick Gel Extraction Kit. The annealed pegRNA sequences were ligated into the vector using T4 DNA ligase. For nickRNA plasmid construction, the pGL3-U6-sgRNA-PGK-puromycin plasmid was digested with BsaI-HFv2 and ligated with the annealed nickRNA using T4 DNA ligase. The ligation products were then transformed into chemically competent E. coli (DH5α). Individual colonies were selected and sequenced to confirm the correct insertion of the pegRNA spacer, scaffold, template, and nickRNA sequences into the respective vectors. All the oligonucleotide sequences can be found in **Supplementary Table 23**.

HEK293T cells were cultured to ~70% confluency. Cells were then passaged and reseeded at a density of 1 × $10^6$ cells per well in a 6-well plate for plasmid transfection. When cells reached ~70% confluency, transfection was performed using Lipofectamine 2000 (Thermo Fisher Scientific) according to the manufacturer's instructions. The transfected plasmids included pCMV-PEmax-P2A-GFP (Addgene ID: 180020), the epgRNA plasmid, and the nickRNA plasmid. After 48 hours post-transfection, GFP-positive cells were sorted using fluorescence-activated cell sorting (FACS) into 96-well plates, with one cell per well. Once single HEK293T clones reached ~70% confluency, cells were passaged, and a portion was collected for genomic DNA extraction. Sanger sequencing was performed to confirm the presence of the intended mutations using the corresponding primers (**Supplementary Table 23**).

Total RNA was extracted using the SteadyPure RNA Isolation Kit according to the manufacturer's instructions. Complementary DNA (cDNA) was synthesized from mRNA using the Hifair® II 1st Strand cDNA Synthesis Kit. Quantitative PCR (qPCR) was then performed using qPCR SYBR Master Mix (Yeasen) following the manufacturer's protocol. The relative proportion between long and short isoforms for each gene were quantified using the corresponding primers (**Supplementary Table 23**).

### Cell line culture

HEK-293T (ATCC, CRL-3216 ™) and SH-SY5Y (ATCC, CRL-2266) were maintained in DMEM (Gibco, C11960500BT), DMEM/F12 (Gibco, C11330500BT) with 10% FBS (Gibco, A5256701) and 1% GlutMAX (Gibco, 35050061). Human iPSC-DYR0100 were purchased from ubigene and cultured in mTeSR plus (stemcell, 100-0276). Cultivated cells were maintained below passage 12 at 37°C with 5% CO2. Media changes and cell passaging were based on recommended schedules and cell confluency. STR confirmed all cell identity. Mycoplasma testing was performed regularly.

### Brain organoid culture

H9 human embryonic stem cells were cultured on Matrigel (Corning, 354277)-coated plates with mTeSR-Plus medium (StemCell Technologies, 100-0276). Cerebral organoids were generated with STEMdiff Cerebral Organoid Kit (StemCell Technologies, 08570) according to the manufacturer's instructions. In brief, on day 0, H9 cells were dissociated into single cells with Gentle Cell Dissociation Reagent (StemCell Technologies, 100-0485). Then 9,000 cells were seeded per well of a 96-well round-bottom ultra-low attachment plate (Corning, 7007) containing 100 μL of EB Seeding Medium, and centrifuged at 300g for 5 min to promote the embryoid bodies (EBs) formation. After 48 hours, 100 μL more of EB Formation Medium were added to each well. On day 3, all of the medium was replaced with 125 μL of Induction Medium. On day 5, each EB was embedded in 25 μL of Matrigel (Corning,

354277). 8-10 embedded organoids were transferred to each well of a 6-well ultra-low adherent plate (Corning, 3471) containing 3 mL of Expansion Medium and incubated for 3 days. On day 8, medium was replaced with 3 mL/well of Maturation Medium. The plate of organoids was moved to an orbital shaker in a 37°C incubator at 60 rpm. Medium was changed every 3 days. The organoid at day 60 was used for fluorescence *in situ* hybridization (FISH) for SNCA 3'UTRs.

### SH-SY5Y and iPSC differentiation

SH-SY5Y cells were differentiated to neuronal cells using a previously reported protocol[3]. Briefly, $3\times10^5$ cells were seeded onto a Matrigel-coated 35mm dish (1:100, Corning, 356231). On the next day, the Stage I differentiation was performed using the medium composed of DMEM supplemented with 2.5% FBS, 10 μM Retinoic acid (MCE, HY-14649), 1×GlutaMax and 1×Penicillin-Streptomycin (Gibco, 15070063). After 5 days, the medium was changed to Stage II differentiation medium composed of Neurobasal™-A (Gibco, 10888022) supplemented with 20mM KCl, 50 ng/ml BDNF(MCE, HY-P7116), 1×B27(Gibco, 17504044), 1×GlutaMax and 1×Penicillin-Streptomycin. The differentiated cells were harvested after 5 days of Stage II differentiation.

For neuron differentiation from iPSC, according to the manufacturer's instructions, we resuspend $3x10^6$ cells in STEMdiff™ Neural Induction Medium (Stemcell, 08581) + SMADi + 10 μM Y-27632 (Selleck, S1049), and then plated into a well of AggreWell™800 24-well plate. During days 1 - 4, daily partial (3/4)-medium changing was performed. On day 5, the cells were plated into a single well of the matrix-coated 6-well plate with EB. From day 6 to day 11, daily full-medium change with prewarmed STEMdiff™ Neural Induction Medium + SMADi was performed. On day 12, the cells with neural rosettes were selected by STEMdiff™ Neural Rosette Selection Reagent (Stemcell, 05832), and were replated to a single well of the matrix-coated 6-well plate. On days 13-17, daily full-medium changing was performed with STEMdiff™ Neural Induction Medium + SMADi until cells were ready for passage. For cell passage, cells were dissociated to single cell with ACCUTASE (Stemcell, 07920), and were then plated onto a new matrix-coated plate with $1x10^6$ cells in 2 mL of STEMdiff™ Neural Progenitor Medium to obtain neural progenitor cells (NPCs). For the differentiation from NPCs to neurons, we aspirated medium and added 2 mL of STEMdiff™ Forebrain Neuron Differentiation Medium (Stemcell, 08600). After 7 days of differentiation, neuron precursors were harvested and seed to PLO/Laminin coated 35mm 4-Chamber glass bottom dish (Cellvis, D35C4) with STEMdiff™ Forebrain Neuron Maturation Medium (Stemcell, 08605) for at least 14 days.

### Gene knockdown in SH-SY5Y-derived neurons

For the validation of potential APA regulators, we performed gene knockdown in SH-SY5Y-derived neurons cells. To construct Dox-inducible shRNA plasmid, shRNA sequences were obtained from the RNAi Consortium collection (Sigma, MISSION® shRNA), and the oligonucleotides were ordered (Sangon) and cloned into pLKO-Tet-On plasmid (Addgene, 21915). All constructs are confirmed by the Sanger sequence. psPAX2 (Addgene, 12260), pMD2.G (Addgene, 12259) and pLKO plasmids were transfected into HEK293T using Lipofectamine™ 3000 (Invitrogen™, L3000015) to produce lentivirus. After 48 hours of transfection, the cell medium was collected and filtered by a 0.45 μm PES filter, and stored at -80°C until use. We then seeded $3\times10^5$ SH-SY5Y cells in a 35mm dish, added 1:100 diluted virus in a 2ml complete medium, and then supplemented 4 μg/mL polybrene. After 6 hours of infection, the medium was changed to fresh medium. After day 3 upon infection,

culture medium was changed to SH-SY5Y Stage I Differentiation Medium (as above). After 5 days of Stage I differentiation, culture medium was changed to Stage II differentiation medium (as above). The differentiated cells were harvested after 5 days of Stage II differentiation, and sent for RNA sequencing.

## RNA-seq of the SH-SY5Y-derived neurons with gene knockdown

We performed RNA-seq of the nuclear RNA fraction to examine the APA alteration upon knockdown of regulator candidates. The oligonucleotides for the knockdown of each gene were available at **Supplementary Table 22.** Total RNA of the SH-SY5Y-derived neuronal cells with gene knockdown was collected according to the manufacturer's instructions (CWbio, CW0591S). RNA quality was assessed on an Agilent 2100 Bioanalyzer (Agilent Technologies) and checked using RNase free agarose gel electrophoresis. mRNA was enriched by Oligo(dT) beads. Then, the enriched mRNA was fragmented using a fragmentation buffer and reversely transcribed into cDNA by NEBNext Ultra RNA Library Prep Kit for Illumina (NEB, 7530). The purified double-stranded cDNA fragments were sent for end repair and dA-tailing, resulting in DNA fragments with 5' phosphorylated and 3' dA-tailed ends. Subsequently, Illumina ligation using NEBNext adaptors were performed. The ligation reaction was purified with the AMPure XP Beads (1.0X), and the PCR amplification was performed. The resulting cDNA library was sequenced using Illumina Novaseq6000 (Repugene Technology, Hangzhou, China).

## Western blots

Protein was purified from the SH-SY5Y neurons using the AllPure DNA/RNA/Protein Kit (CWbio, CW0591S) and dissolved in Cell lysis buffer (Beyotime, P0013) supplemented with 1x protease inhibitor (Roche, 4693116001). Protein concentration was determined by BCA Protein Assay Kit (Beyotime), and then an equal volume of 2x loading buffer was added and incubated at 95°C for 5 minutes. A total of 10μg of each protein sample was separated on a 4-20% SurePAGE precast mini polyacrylamide gels (GenScript, M00657) and transferred onto nitrocellulose membranes (Cytiva,10600001). Membranes were incubated with 5% BSA blocking buffer, then incubated with primary antibodies, and left overnight at 4°C. After incubation in a secondary antibody (Invitrogen, 31430/31460) for 2h at room temperature, the targeted proteins were detected using ECL Chemiluminescence kit (Vazyme, E422). Then the loading control protein Histone H3 (proteintech, 17168-1-AP) was measured on the same membrane as a control to account for the total protein content.

## Separation of Nuclear and cytoplasmic RNA

Nuclear and cytoplasmic RNA were isolated according to the manufacturer's instructions (Vazyme, E101). Briefly, SH-SY5Y-differentiated neuron cells were resuspended in Cytoplasm Extraction Buffer A supplemented with RNase inhibitor (Vazyme, R301) and incubated on ice for 15 minutes. Cytoplasm Extraction Buffer B was added, and the mixture was vortexed and incubated for 4 minutes. The mixture was then centrifuged at 17,000 x g for 30 seconds, and the supernatant (cytoplasmic RNA) was transferred to a new tube and collected. The remaining pellet (nuclear fraction) was resuspended in PBS supplemented with RNase inhibitor and immediately centrifuged at 17,000 x g for 30 seconds. The resulting pellet (nuclear RNA) was collected.

## Nuclear versus cytoplasmic abundance of 3'UTR isoforms

To examine the relative abundance of long versus short 3'UTR isoforms in nuclear versus cytoplasmic fractions for AD-DAPAs and *SNCA*, we conducted quantitative real-time PCR

(qPCR) experiments long and short isoforms in SH-SY5Y-differentiated neurons based on the abovenucleus-cytoplasm separation experiments. The primers for the distal and proximal APA isoforms of each gene are included in **Supplementary Table 24.**

First, we collected the RNA from nuclear and cytoplasmic fractions, according to the manufacturer's instructions (CWbio, CW0591S). Then, for each fraction, 200ng RNA were used for reverse transcription with TransScript® Uni All-in-One First-Strand cDNA Synthesis SuperMix (Transgen, AU341). We next conducted qPCR experiments to quantify the relative expression of all genes for the two fractions with two technical replicates. Specifically, we used a reaction mix containing ChamQ Universal SYBR qPCR Master Mix (Vazyme, Q711) to conduct qPCR experiment, then used the LC480 system (Roche) to measure the fluorescence at each cycle.

For the quantification of relative nuclear/cytoplasmic ratio, the calculation was carried out in the following steps: 1) compute the relative expression quantified by the primers targeting the distal-pA-isoform-specific and shared 3'UTR regions in the nuclear and cytoplasmic fractions, respectively, normalizing each to *GAPDH* expression within each corresponding fraction; 2) calculate the fold change of the normalized expression between nucleus and cytoplasm for the shared and long isoforms of each gene; 3) for each gene, compare its relative nuclear/cytoplasmic ratio between the shared versus the long isoform, and used a paired Wilcoxon test to perform significance tests. To ensure the quality of fraction separation experiments, we examined the expression of *GAPDH*, *ACTIN* and *XIST*, where *XIST* was a nucleus-specific marker RNA.

### *In situ* RNA location detection using FISH

To estimate the effect of APAs on the relative abundance in nucleus versus cytoplasm, we carried out an *in situ* RNA location detection assay. For oligonucleotide sequence design, the fasta sequences of shared- and differential-3'UTR regions were extracted by the getfasta function of bedtools (v2.26.0)[4]. We designed three oligonucleotide sequences for each region of all tested APAs (**Supplementary Table 25**).

Cultured brain organoid was firstly placed in O.C.T and immersed in snap-cold liquid nitrogen for 2 minutes, then either stored at -80℃ or cut as 12-μm slices using a cryostat (Leica). Slices were fixed with 2% (w/v) paraformaldehyde in PBS at room temperature (r.t.) for 5 min, then permeabilized with 0.1 M HCl at r.t. for 5 min, washed twice in DEPC-treated PBS (DEPC-PBS) and dehydrated through a series of 70%, 85% and 100% ethanol for 2 min each. Then, RNA *in situ* detection was performed using the described protocol[5], with the detailed procedure as follows. Firstly, reverse transcription was carried out overnight to synthesize in situ cDNA. Secondly, padlock probes ligating at the target sequence were specifically hybridized to the target cDNA. Thirdly, the ligated padlock probes were amplified through rolling-circle amplification (RCA) at the site, and Φ29 DNA polymerase (Fermentas) was used for this amplification reaction. Fourth, the single-stranded DNA obtained from PCA was hybridized by fluorescently labeled detection probes for further visualization. Finally, we used a fluorescence microscope (Zeiss LSM900) to acquire the images and used ZEN3.10 software to quantify the ratio of nuclear-located mRNA molecules. In total, seven brain organoids cultured in three differentiation batches were utilized for the experiment. For each organoid, we averaged measurements from three randomly selected fields of view (20×) from one slice of the organoid. The number of fluorescent spots that overlapped with DAPI fluorescence and extra-nuclear were counted. We used the paired Wilcoxon test to estimate

the difference of nuclear-RNA-ratio between shared- and differential-3'UTR groups for each APA gene.

## Single-molecule inexpensive FISH (smiFISH) for subcellular localization investigation

Each primary probe was designed to contain a gene-specific sequence targeting the RNA region of interest and an additional 18-nucleotide universal sequence (5′–tgcaggtcgactctagaa–3′) at its 5′ end, as originally described by Bertrand *et al.*[6]. The universal secondary probe carried the complementary sequence to this 18-nt tag and was labeled with ROX fluorophores at both ends (TsingkeBiotechnologyCo.,Ltd.). All the oligonucleotide probes used in smiFISH experiments are included in **Supplementary Table 25.**

For each gene, two independent batches of iPSC-differentiated neurons were used for experiments. Cells were washed once with phosphate-buffered saline (PBS) and fixed in 4% paraformaldehyde (PFA) at 37 °C for 10 min. Fixed cells were washed twice with PBS (5 min each) and permeabilized in 70% ethanol (400 µl per well, diluted in RNase-free water) at 4 °C for 8 h. Before hybridization, cells were equilibrated in wash buffer (2× saline sodium citrate [SSC, Invitrogen,155570440], 10% formamide [Coolaber, CF5401] in RNase-free water) at 37 °C for 5 min.

**Primary hybridization:** For each well, 200 µl of primary hybridization mix was added and incubated at 37 °C for 16 h in the dark. After hybridization, cells were washed twice with pre-warmed wash buffer at 37 °C (15 min each, protected from light).

**Secondary hybridization:** A secondary hybridization mix (200 µl per well) containing the fluorescently labeled universal probes was added and incubated for an additional 6 h at 37 °C in the dark. Cells were then washed twice with a pre-warmed with pre-warmed wash buffer (400 µl per well, 15 min each) at 37 °C in the dark, followed by a quick rinse with PBS before imaging.

The hybridization buffer consisted of 5% dextran sulfate (Coolaber, CD4411), 10% formamide, 2× SSC, 2 mM ribonucleoside vanadyl complex (New England Biolabs, S1402S), 200 µg $ml^{-1}$ yeast tRNA (Sigma, 10109495001), and 100 nM probe mixture (primary or secondary probes). All procedures were performed under RNase-free conditions and protected from light.

**Imaging and Quantification:** Fluorescence imaging was performed using an Olympus IXplore SpinSR microscope equipped with a Yokogawa CSU-W1 SoRa spinning-disk confocal unit and a 60× oil immersion objective. The system was operated in super-resolution (SR) mode to achieve an enhanced spatial resolution for individual RNA molecules. Z-stack images were acquired with an optimal step size to capture single-molecule signals throughout the cell volume. Acquisition parameters, including laser intensity and detector gain, were kept constant across samples to ensure quantitative comparability.

Image analysis was conducted using *Imaris* software (Bitplane). Individual RNA molecules were automatically detected as discrete fluorescent puncta using the *Spots* module with consistent threshold parameters applied to all datasets. Detected spots were visually inspected to confirm accurate segmentation and to remove background artifacts. The

number and spatial distribution of RNA molecules per cell were extracted for quantitative analysis.

## References


1. Danecek, P. *et al.* Twelve years of SAMtools and BCFtools. *Gigascience* **10**, (2021).
2. Doman, J. L., Sousa, A. A., Randolph, P. B., Chen, P. J. & Liu, D. R. Designing and executing prime editing experiments in mammalian cells. *Nat Protoc* **17**, 2431–2468 (2022).
3. Dravid, A., Raos, B., Svirskis, D. & O'Carroll, S. J. Optimised techniques for high-throughput screening of differentiated SH-SY5Y cells and application for neurite outgrowth assays. *Sci Rep* **11**, 23935 (2021).
4. Quinlan, A. R. & Hall, I. M. BEDTools: a flexible suite of utilities for comparing genomic features. *Bioinformatics* **26**, 841–842 (2010).
5. Larsson, C., Grundberg, I., Söderberg, O. & Nilsson, M. In situ detection and genotyping of individual mRNA molecules. *Nat. Methods* **7**, 395–397 (2010).
6. Bertrand, E. *et al.* Localization of ASH1 mRNA particles in living yeast. *Mol. Cell* **2**, 437–445 (1998).
