# Supplementary material for: Single-cell profiling and genetic regulation of alternative polyadenylation in the human brain
Source: Nat Genet. 2026 Sep 30;58(11):2819–31. doi: 10.1038/s41588-026-02758-w (PMC13754597; doi:10.1038/s41588-026-02758-w)
Supplement: Supplementary file 2 [file 41588_2026_2758_MOESM2_ESM.pdf]

nature portfolio

Corresponding author(s): Xushen Xiong, Carles Boix

Last updated by author(s): Jun 25, 2026

Reporting Summary

Nature Portfolio wishes to improve the reproducibility of the work that we publish. This form provides structure for consistency and transparency in reporting. For further information on Nature Portfolio policies, see our Editorial Policies and the Editorial Policy Checklist.

Statistics

For all statistical analyses, confirm that the following items are present in the figure legend, table legend, main text, or Methods section.

n/a	Confirmed	
☐	☒	The exact sample size (*n*) for each experimental group/condition, given as a discrete number and unit of measurement
☐	☒	A statement on whether measurements were taken from distinct samples or whether the same sample was measured repeatedly
☐	☒	The statistical test(s) used AND whether they are one- or two-sided<br>*Only common tests should be described solely by name; describe more complex techniques in the Methods section.*
☐	☒	A description of all covariates tested
☐	☒	A description of any assumptions or corrections, such as tests of normality and adjustment for multiple comparisons
☐	☒	A full description of the statistical parameters including central tendency (e.g. means) or other basic estimates (e.g. regression coefficient) AND variation (e.g. standard deviation) or associated estimates of uncertainty (e.g. confidence intervals)
☐	☒	For null hypothesis testing, the test statistic (e.g. *F*, *t*, *r*) with confidence intervals, effect sizes, degrees of freedom and *P* value noted<br>*Give P values as exact values whenever suitable.*
☒	☐	For Bayesian analysis, information on the choice of priors and Markov chain Monte Carlo settings
☒	☐	For hierarchical and complex designs, identification of the appropriate level for tests and full reporting of outcomes
☐	☒	Estimates of effect sizes (e.g. Cohen's *d*, Pearson's *r*), indicating how they were calculated

Our web collection on statistics for biologists contains articles on many of the points above.

Software and code

Policy information about availability of computer code

Data collection

no software was used

Data analysis

All the code is available at https://github.com/xiongxslab/NanJ_Carles_et_al_3aQTL_project/.
Identification of pA sites and transcriptome-wide 3’ UTR length comparison
We carried out de novo pA site identification at single-cell level using SCAPE, a Bayesian method that models and identifies pA sites based on a Gaussian distribution107. To be consistent with the snRNA-seq reads alignment, we utilized GENCODE (Version 32) gene annotation of the GRCh38 genome for identifying pA sites. The identified pA sites were first validated by showing the motif enrichments of the flanking sequences. Specifically, we utilized DREME (Version 5.5.4) to search for enriched motifs within the 50 bp sequences upstream of the pA sites, and the identified A[A/T]TAAA motif was subsequently used as an input in FIMO to locate the position of the motif relative to the pA sites108. For the comparison of global 3’ UTR length across all the genes, we first computed the 3’ UTR length for each pA site by calculating the distance between the pA site and the annotated stop codon with introns excluded. For each gene in each cell, we calculated a mean 3’ UTR length across all the pA sites of that gene, weighted by the supporting counts of each pA site. We then calculated a “scaled 3’ UTR length” for each gene in each cell, by subtracting the 3’ UTR length in that cell by the mean of 3’ UTR lengths across all the cells. Subsequently, for each cell the global 3’ UTR length can be calculated as the mean of the scaled 3’ UTR length across all the genes in that cell. Such scaled 3’ UTR length calculation is based on the relative length distribution across cells, therefore enables the comparison of 3’ UTR length between different genes and cells. We further defined the 3’ UTR length at the pseudo-bulk level by calculating the mean 3’ UTR length across all the cells in each cell type from each individual to ensure the robustness for the analyses of 3’ UTR comparison across cell types and individuals, as well as the identification of APA regulators.
Two-pA-site-based quantification of APA
To facilitate 3’aQTL identification and differential APA analyses, we utilized a well-established two-pA-site model based on DaPars215. We first generated pseudo-bulk alignment files for each cell type in each sample, based on the major cell type cell annotation14. For each gene in each

pseudo-bulk sample, we identified proximal and distal pA sites using DaPars2 and then calculated a PDUI (percentage of distal poly-A site usage index), which is defined as the ratio between number of distal reads and the sum of proximal and distal reads. A larger value of PDUI represents a larger fraction of the long version of 3' UTR.

For manuscripts utilizing custom algorithms or software that are central to the research but not yet described in published literature, software must be made available to editors and reviewers. We strongly encourage code deposition in a community repository (e.g. GitHub). See the Nature Portfolio guidelines for submitting code & software for further information.

Data

Policy information about availability of data

All manuscripts must include a data availability statement. This statement should provide the following information, where applicable:

- Accession codes, unique identifiers, or web links for publicly available datasets
- A description of any restrictions on data availability
- For clinical datasets or third party data, please ensure that the statement adheres to our policy

Data availability
The snRNA-seq data are available at Synapse under the accession syn52293417. The summary statistics for the 3'aQTLs and the eQTLs generated by this study are made available at https://cboix.github.io/snAPA and at Zenodo https://zenodo.org/records/20740673. The RNA-seq data of the knockdown experiments for regulator validation are available at National Genomics Data Center (NGDC) under the accession HRA014304.

Research involving human participants, their data, or biological material

Policy information about studies with human participants or human data. See also policy information about sex, gender (identity/presentation), and sexual orientation and race, ethnicity and racism.

Reporting on sex and gender	We used 379 individuals from the Religious Orders Study and Rush Memory and Aging Project (ROSMAP), both ongoing longitudinal clinical-pathologic cohort studies of aging and dementia, in which all participants are brain donors. Individuals were balanced between sexes (male:female ratios 188:191) (sex was determined based on self-reporting).
Reporting on race, ethnicity, or other socially relevant groupings	No socially constructed or socially relevant categorization variables were used in this study.
Population characteristics	We used 379 individuals from the Religious Orders Study and Rush Memory and Aging Project (ROSMAP), both ongoing longitudinal clinical-pathologic cohort studies of aging and dementia, in which all participants are brain donors. For the purpose of this study, individuals were selected based on the modified NIA-Reagan diagnosis of Alzheimer's disease, with nonAD vs AD ratio 165:214.
Recruitment	No donors were recruited, the tissue has been obtained from participants in the Religious Order Study.
Ethics oversight	The Religious Orders Study and Rush Memory and Aging Project were approved by an IRB of Rush University Medical Center.

Note that full information on the approval of the study protocol must also be provided in the manuscript.

Field-specific reporting

Please select the one below that is the best fit for your research. If you are not sure, read the appropriate sections before making your selection.

☒ Life sciences ☐ Behavioural & social sciences ☐ Ecological, evolutionary & environmental sciences

For a reference copy of the document with all sections, see nature.com/documents/nr-reporting-summary-flat.pdf

Life sciences study design

All studies must disclose on these points even when the disclosure is negative.

Sample size	For the brain snRNA-seq samples, the data were collected from a published study.<br>For the experiments performed in our study, the sample sizes were selected based on established practice in the field. For RNA-seq gene knockdown experiments, independent biological replicates with different shRNAs were performed for each target to ensure reproducibility and enable statistical assessment of differential expression and APA changes. For prime-editing validation of 3' aQTLs, multiple independently edited clones were generated to span a range of editing efficiencies, allowing robust assessment of the relationship between genotype and APA. For single-molecule FISH (smiFISH) experiments, independent batches of differentiated neurons were analyzed, and multiple fields of view and large numbers of cells were quantified from each batch to provide sufficient statistical power.<br>All experiments showed consistent effects across independent replicates, and the resulting sample sizes were sufficient to detect the reported differences based on the rigorous statistical tests, as described in the Methods and figure legends.
Data exclusions	Low quality snRNA-seq libraries were excluded and the exclusion criteria are described in the manuscript as follows. We kept only protein coding genes and filtered out cells with over 20% mitochondrial or 5% ribosomal RNA, leaving 1.47M cells over 48 individuals and 283 samples

across all regions. We separately called doublets using DoubletFinder and flagged and removed clusters with strong doublet profiles and clusters showing strong individual-specific batch effects, leaving a final dataset of 1.35M cells.

Replication	For gene knockdown experiments, both independent biological replicates using different shRNAs and technical replicates were performed for each target. Knockdown efficiency and downstream effects were assessed by western blot, qPCR, and RNA-seq across these replicates. For single-molecule FISH (smiFISH) experiments, independent batches of differentiated neurons were analyzed, and multiple fields of view (FOVs) were sampled from each batch. Quantitative analyses were performed using the indicated biological replicates and appropriate statistical tests, as described in the Methods and figure legends.
Randomization	Random allocation was not relevant to this study. Experimental groups were predefined by study design, including control vs gene knockdown, edited vs wild-type for 3'aQTL validation, and disease or cell-type classifications for snRNA-seq analyses. For snRNA-seq analyses, potential confounding variables were accounted for in the statistical analyses where appropriate (for example, age, sex, and post-mortem interval in differential APA analyses), as described in the Methods.
Blinding	Blinding was not relevant to this study.<br>For AD vs control group allocation, the snRNA-seq was collected from a published study (as described in the Methods).<br>For analyses, quantification for all experiments was performed using standardized computational pipelines.

Reporting for specific materials, systems and methods

We require information from authors about some types of materials, experimental systems and methods used in many studies. Here, indicate whether each material, system or method listed is relevant to your study. If you are not sure if a list item applies to your research, read the appropriate section before selecting a response.

Materials & experimental systems

n/a	Involved in the study
☐	☒ Antibodies
☐	☒ Eukaryotic cell lines
☒	☐ Palaeontology and archaeology
☒	☐ Animals and other organisms
☒	☐ Clinical data
☒	☐ Dual use research of concern
☒	☐ Plants

Methods

n/a	Involved in the study
☒	☐ ChIP-seq
☒	☐ Flow cytometry
☒	☐ MRI-based neuroimaging

Antibodies

Antibodies used

(Protein, Manufacturer, catalog number, lot number, working dilution, validation information for each antibody)

Immunofluorescence:
PAX6, BioLegend, 901302, B354381, 1:2000, Vendor information;
MAP2, Abcam, AB5392, 1057905-3, 1:5000, Vendor information;
Beta-Tubulin III, Stemcell, 60052, 1000142927, 1:2000, Vendor information;
Goat Anti-Mouse IgG H&L (Alexa Fluor® 568), ab175473, 1052446-1, 1:2000, Vendor information;
Goat Anti-Chicken IgY H&L (Alexa Fluor® 647), ab150171, 1022461-6, 1:2000, Vendor information;
Goat Anti-Rabbit IgG H&L (Alexa Fluor® 488), ab150077, 1052444-1, 1:2000, Vendor information;

Western Blot:
PABPN1, ProteinTech, 66807-1-Ig, 10007344, 1:2000, Vendor information;
PCBP2, ProteinTech, 15070-1-AP, 00043756, 1:6000, Vendor information;
U2AF2, ProteinTech, 15624-1-AP, 00039254, 1:2000, Vendor information;
PNKD, Huabio, ER61038, H640160015, 1:1000, Vendor information;
GLYR1, ProteinTech, 14833-1-AP, 00185285, 1:1000, Vendor information;
ATL2, ProteinTech, 16688-1-AP, 00053330, 1:1000, Vendor information;
TOLLIP, ProteinTech, 11315-1-AP, 00143372, 1:1000, Vendor information;
H3, ProteinTech, 17168-1-AP, 00172675, 1:5000, Vendor information;
Goat anti-Rabbit IgG (H+L) HRP, Invitrogen, 31460, XI363512, 1:100000, Vendor information;
Goat anti-Mouse IgG (H+L) HRP, Invitrogen, 31430, XI363703, 1:100000, Vendor information;

Validation

All the antibodies were commercially available with validation done by the manufacturer.

Eukaryotic cell lines

Policy information about cell lines and Sex and Gender in Research

Cell line source(s)	SH-SY5Y (female), Ubigene, YC-D014; iPSC (male), Ubigene, YC-C098; H9 (female), From Professor Xudong Fu's Lab.
Authentication	All cell lines were confirmed to match original line by STR fingerprinting.

Mycoplasma contamination	We confirm that the cell line we used was tested negative for mycoplasma contamination.
Commonly misidentified lines (See ICLAC register)	We did not use commonly misidentified lines.

Plants

Seed stocks	*Report on the source of all seed stocks or other plant material used. If applicable, state the seed stock centre and catalogue number. If plant specimens were collected from the field, describe the collection location, date and sampling procedures.*
Novel plant genotypes	*Describe the methods by which all novel plant genotypes were produced. This includes those generated by transgenic approaches, gene editing, chemical/radiation-based mutagenesis and hybridization. For transgenic lines, describe the transformation method, the number of independent lines analyzed and the generation upon which experiments were performed. For gene-edited lines, describe the editor used, the endogenous sequence targeted for editing, the targeting guide RNA sequence (if applicable) and how the editor was applied.*
Authentication	*Describe any authentication procedures for each seed stock used or novel genotype generated. Describe any experiments used to assess the effect of a mutation and, where applicable, how potential secondary effects (e.g. second site T-DNA insertions, mosiacism, off-target gene editing) were examined.*
