# Supplementary material for: Single-cell profiling and genetic regulation of alternative polyadenylation in the human brain
Source: Nat Genet. 2026 Sep 30;58(11):2819–31. doi: 10.1038/s41588-026-02758-w (PMC13754597; doi:10.1038/s41588-026-02758-w)
Supplement: Supplementary file 3 [file 41588_2026_2758_MOESM5_ESM.pdf]

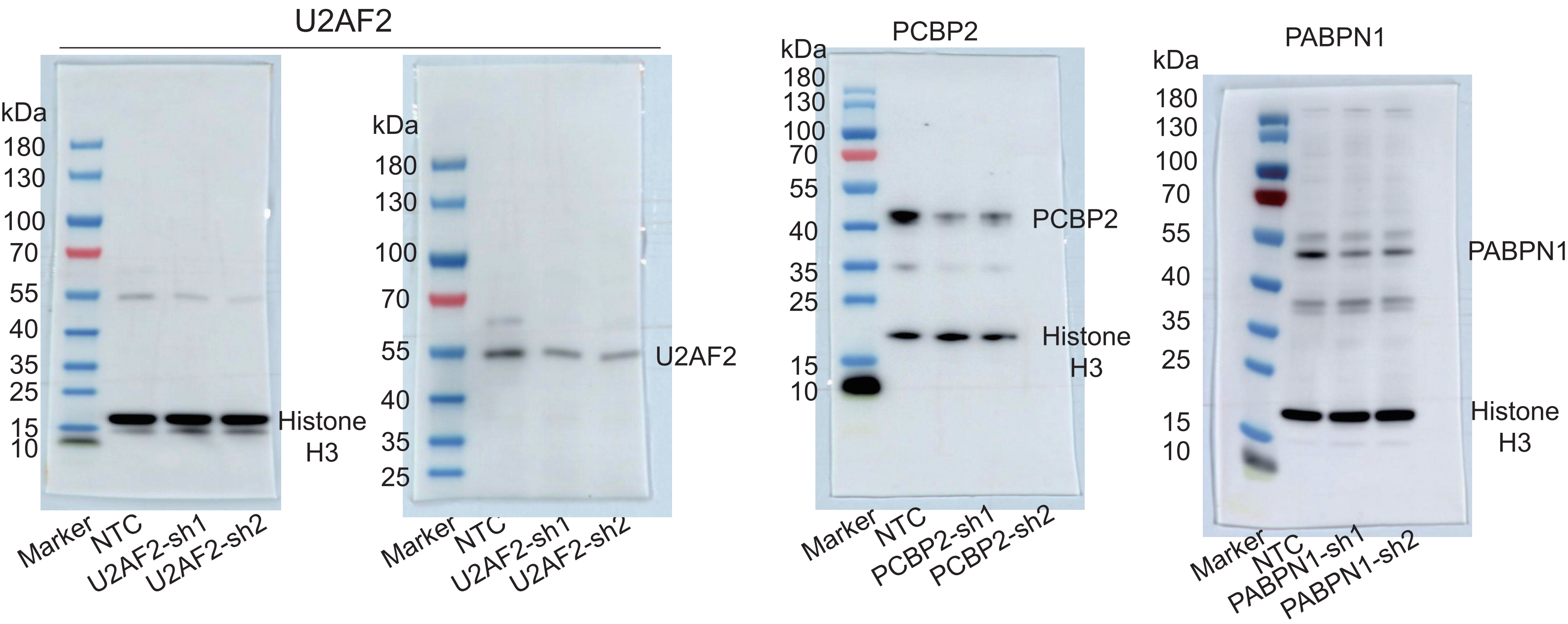
U2AF2
kDa
180
130
100
70
55
40
35
25
15
10
Histone
H3
Marker
NTC
U2AF2-sh1
U2AF2-sh2
kDa
180
130
100
70
55
40
35
25
U2AF2
Marker
NTC
U2AF2-sh1
U2AF2-sh2
PCBP2
kDa
180
130
100
70
55
40
35
25
15
10
PCBP2
Histone
H3
Marker
NTC
PCBP2-sh1
PCBP2-sh2
PABPN1
kDa
180
130
100
70
55
40
35
25
15
10
PABPN1
Histone
H3
Marker
NTC
PABPN1-sh1
PABPN1-sh2
